# Venetoclax imparts distinct cell death sensitivity and adaptivity patterns in T cells

**DOI:** 10.1038/s41419-021-04285-4

**Published:** 2021-10-27

**Authors:** Lindsey M. Ludwig, Katrina M. Hawley, David B. Banks, Anika T. Thomas-Toth, Bruce R. Blazar, Megan E. McNerney, Joel D. Leverson, James L. LaBelle

**Affiliations:** 1 Department of Pediatrics, Section of Hematology/Oncology, University of Chicago, Chicago, IL USA; 2 Medical Scientist Training Program, University of Chicago, Chicago, IL USA; 3 Department of Pediatrics, Division of Blood and Marrow Transplantation, University of Minnesota, Minneapolis, MN USA; 4 Department of Pathology, University of Chicago, Chicago, IL USA; 5 AbbVie Inc., North Chicago, IL USA

**Keywords:** Bone marrow transplantation, Immune cell death

## Abstract

BH3 mimetics are increasingly used as anti-cancer therapeutics either alone or in conjunction with other chemotherapies. However, mounting evidence has also demonstrated that BH3 mimetics modulate varied amounts of apoptotic signaling in healthy immune populations. In order to maximize their clinical potential, it will be essential to understand how BH3 mimetics affect discrete immune populations and to determine how BH3 mimetic pressure causes immune system adaptation. Here we focus on the BCL-2 specific inhibitor venetoclax (ABT-199) and its effects following short-term and long-term BCL-2 blockade on T cell subsets. Seven day “short-term” ex vivo and in vivo BCL-2 inhibition led to divergent cell death sensitivity patterns in CD8^+^ T cells, CD4^+^ T cells, and Tregs resulting in shifting of global T cell populations towards a more memory T cell state with increased expression of BCL-2, BCL-X_L_, and MCL-1. However, twenty-eight day “long-term” BCL-2 blockade following T cell-depleted bone marrow transplantation did not lead to changes in the global T cell landscape. Despite the lack of changes in T cell proportions, animals treated with venetoclax developed CD8^+^ and CD4^+^ T cells with high levels of BCL-2 and were more resistant to apoptotic stimuli following expansion post-transplant. Further, we demonstrate through RNA profiling that T cells adapt while under BCL-2 blockade post-transplant and develop a more activated genotype. Taken together, these data emphasize the importance of evaluating how BH3 mimetics affect the immune system in different treatment modalities and disease contexts and suggest that venetoclax should be further explored as an immunomodulatory compound.

## Introduction

While the clinical use of BH3 mimetics expands against a myriad of cancers [1, 2], little is known about how these compounds affect healthy immune cells [3–5]. A growing body of evidence demonstrates that leukocytes are affected by BH3 mimetics, at doses equivalent to or lower to those typically used in the context of cancer therapy [5–7]. Our goal in this study was to gain a greater understanding of how surviving immune cells adapt to venetoclax, a BH3 mimetic specific for BCL-2, with a particular focus on T cells whose apoptotic dependency varies depending on lineage and effector function [8–10].

We previously found that CD4^+^ and CD8^+^ T cells adapt differently to the loss of BIM, the dominant pro-apoptotic BH3-only protein in T cells [11]. Herein we aimed to determine how therapeutic BCL-2 blockade, rather than deletion of its major BH3-only binding partner, alters the T cell compartment. We found that CD8^+^, CD4^+^, and regulatory (Treg) T cells have disparate cell death sensitivities to venetoclax at baseline and developed varying anti-apoptotic protein levels and cell death resistance when expanded in the presence of venetoclax. In vivo, as a whole, CD8^+^ T cells were most sensitive to short-term venetoclax treatment while Tregs were most resistant. T cell death patterns reflected their activation state, with naïve T cells being more sensitive than memory cells, supporting other recent pre-clinical observations in the context of anti-tumor T cell responses [4]. However, this was not the case for long-term treatment through T cell engraftment following autologous bone marrow transplantation. In this setting, donor-derived T cells overcame BCL-2 blockade to normalize their overall ratios. However, despite a normal appearing phenotypic repertoire, venetoclax resulted in altered anti-apoptotic protein levels and increased cell death resistance to a number of apoptotic stimuli. Surprisingly, venetoclax altered the mRNA expression profile of naive CD4^+^ and CD8^+^ T cells post-transplant towards an “activated-like” state particularly through the upregulation of Jak-STAT and downregulation of MAPK and FoxO signaling pathways. Overall, these data expand our understanding of T cell acclimation or adaptivity in response to BCL-2 targeting, which may have important implications for the expanded clinical use of venetoclax and other BH3 mimetics.

## Materials and methods

### Animals

B6.Cg-*Foxp3*^*tm2Tch*^*/*J (FOXP3-IRES-GFP) [12] mice were gifts from Maciej Lesniak, M.D. CD45.1 C57BL/6 and WT (CD45.2) C57BL/6 mice were purchased from The Jackson Labs. Compete blood counts were measured from the peripheral blood of animals using the IDEXX Procyte Dx Hematology Analyzer (IDEXX Laboratories, Westbrook, ME). Animal experiments were approved by and performed in accordance with the guidelines and regulations set forth by the Institutional Animal Care and Use Committee of the University of Chicago.

### Cell sorting and flow cytometric analysis

Single cell suspensions were generated as previously described [13]. Sorted populations used for analysis were ≥97% pure. In all cases, Tregs were sorted and analyzed separately from conventional CD4^+^ T cells by virtue of their expression of GFP and also confirmed by intracellular expression of FOXP3. A full list of antibodies used for immune cell phenotyping can be found in the supplementary information. Intracellular protein staining was performed with the Fixation/Permeabilization kit (eBioscience, San Diego, CA) per the manufacturer’s protocol. Cells were stained with FOXP3-APC(FJK-16s), BCL-2-PE(3F11), BIM-PE(C34C5), BCL-X_L_-PE(54H6), and MCL-1-PE(D2W9E). Intracellular flow cytometric detection of BCL-2 family proteins was performed as previously described [14]. Samples were analyzed at the University of Chicago Flow Cytometry Core using a LSRII (BD, Franklin Lakes, NJ) or FACSAria (BD). Data analysis was performed using FlowJo (Tree Star, BD).

### T cell stimulation, expansion, and BH3 profiling

CD8^+^ T cells, CD4^+^FOXP3^−^ T cells (CD4^+^ T cells), and CD4^+^FOXP3^+^GFP^+^ T cells (Tregs) were cultured as previously described [11] and expanded using CD3/CD28 Dynabeads (Life Technologies, Carlsbad, CA) at a 1:1 bead:cell ratio and 500 U/mL of recombinant IL-2 (Prometheus Therapeutics & Diagnostics, San Diego, CA) (CD8^+^ and CD4^+^ T cells) or with a 3.5:1 bead:cell ratio and 2,000 U/mL IL- 2 (Tregs). Cells were treated with DMSO (vehicle) or 500 nM venetoclax (AbbVie, North Chicago, IL) daily for five days after reaching logarithmic growth and >95% viable (day 3). BH3 profiling was performed as previously described [11]. % Depolarization was calculated using the following formula: % Depolarization = (1−(sample-FCCP)/(DMSO-FCCP)) × 100.

### Long-term venetoclax treatment

CD45.1^+^ C57BL/6 recipient mice were conditioned using total body gamma irradiation (Cesium-137) at a fractionated dose of 2x 550 cGy separated by 3 h (1100 cGy total). 24 h later, mice were transplanted intravenously with 2 × 10^6^ T cell-depleted (TCD) bone marrow cells harvested from the femurs and tibia of CD45.2^+^ FOXP3-IRES-GFP donor mice as previously described [15]. TCD was performed using anti-CD3 mAb coated magnetic beads (MACS separation, Miltenyi, Bergisch Gladbach, Germany). Venetoclax was administered via oral gavage at doses of 25 mg/kg, 50 mg/kg, or 100 mg/kg as previously described [13, 16, 17].

### T cell viability measurement

T cells were transferred to 96-well round bottom plates (Denville, South Plainfield, NJ) at a density of 5 × 10^4^ T cells/well (sorted T cells) or 2 × 10^5^ splenocytes/well (following transplantation) and treated with increasing concentrations of venetoclax. Cells were cultured in media alone (cDMEM, cytokine deprivation), 1 μg/mL ionomycin, 4 ng/mL PMA, 1 μM etoposide, 250 nM SAHA/vorinostat, and 50 nM staurosporin. Following 24 h incubation, cell death was assessed using Annexin V-APC (Life Technologies) and propidium iodide (Life Technologies) as previously reported [18].

### Reverse transcription and quantitative PCR

Cells were lysed with Trizol (Life Technologies) and mRNA extracted using the Direct-zol RNA MiniPrep kit (Zymo, Irvine, CA). mRNA concentration and purity were assessed using a DeNovix DS-11 spectrophotometer and reverse transcription performed using the Superscript III first strand synthesis reverse transcription kit (Invitrogen, Carlsbad, CA) per the manufacturer’s guidelines. Reverse transcription and quantitative PCR was performed using TaqMan Master Mix and Gene Expression Probes (Applied Biosystems, Foster City, CA) as detailed in the supplementary information. Samples were run on the 7500 Fast Real-Time PCR System (Applied Biosystems). Data was analyzed with the ExpressionSuite software using the ∆∆CT method with UBC as the housekeeping gene and untreated or unstimulated T cells for the reference samples.

### RNA-seq

Library preparation and sequencing were performed by the University of Chicago Genomics Facility. 30 million, paired end, 100 bp reads for each sample were generated using the NovaSeq6000 (Illumina, San Diego, CA). Alignment to the murine genome (mm10) was performed using HISAT2. Differential expression analysis was performed using EdgeR, and data was filtered to exclude genes with counts per million (CPM) <2 in 3 or more samples [19]. Gene set enrichment analysis was performed as described previously [20]. Significantly upregulated and downregulated genes (*p* < 0.01) were analyzed separately for enrichment of KEGG pathways using the DAVID bioinformatics platform with an FDR threshold of <0.05 [21, 22].

### Statistical analysis

A one-way ANOVA test was used to evaluate statistically significant changes for increasing doses of venetoclax. For all other comparisons, an unpaired Student’s *t* test was performed and data are expressed as the means ± SEM. All in vivo treatment experiments were performed a minimum of three separate times. Cells collected from mice were analyzed separately (*i.e*. not pooled) thereby allowing for biological replicates. Plots were created using Prism (GraphPad Software, La Jolla, CA, USA). Statistical significance was defined as *p < 0.05, **p < 0.01, ***p < 0.001, ****p < 0.0001.

## Results

### Ex vivo expansion in the presence of venetoclax distinctly alters cell death resistance in different T cell subtypes

**Fig. 1:**
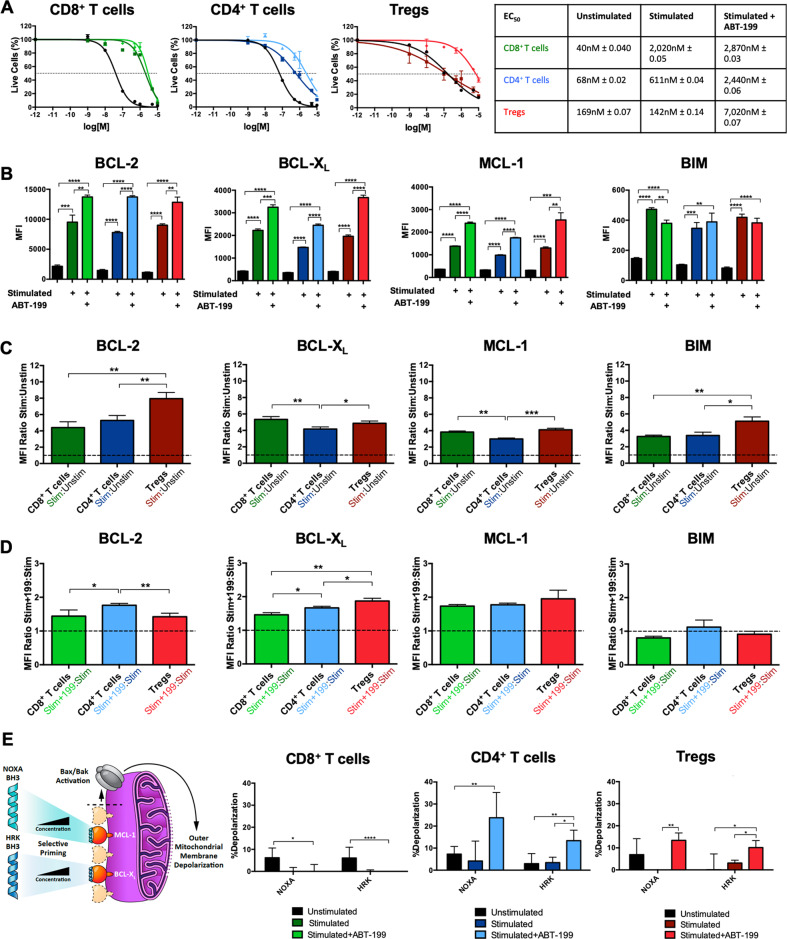
Ex vivo stimulation and expansion of CD8^+^ T cells, CD4^+^ T cells, and Tregs alone or in the setting of BCL-2 blockade results in divergent cell death resistance and BCL-2 family protein expression. **A** Cell death of CD8^+^ T cells, CD4^+^ T cells, and Tregs treated with venetoclax directly following isolation and after stimulation/expansion alone or in the presence of venetoclax. **B** BCL-2 family protein levels of unstimulated and stimulated/expanded T cells alone or in the presence of venetoclax. **C** Ratios of BCL-2 family protein levels in stimulated/expanded T cells compared to unstimulated and **D** between stimulated/expanded in the presence of venetoclax compared to stimulated/expanded alone. **E** BH3 profiling of T cells using peptides specific for MCL-1 (NOXA) and BCL-X_L_ (HRK) to determine shifts in anti-apoptotic dependencies. Schematic of BH3-peptide specificity and ability to induce mitochondrial outer membrane permeabilization (MOMP) is shown on the left-hand side. Data represented in black represents treatment of cells prior to stimulation. Colored data (CD8^+^ T cells in green, CD4^+^ T cells in blue, and Tregs in red) represent treatment following stimulation (dark green, blue, and red) or following stimulation in the presence of venetoclax (light green, blue, and red). Data is representative of three independent replicates. Data represented as means ± SEM. *p < 0.05, **p < 0.01, ***p < 0.001, ****p < 0.0001.

To first determine if venetoclax-induced T cell-specific “adaptive” differences, we measured sensitivity to venetoclax in freshly isolated cells ex vivo. CD8^+^ and CD4^+^ T cells showed similar baseline sensitivities to venetoclax (EC_50_ 40 nM and 68 nM respectively) while Tregs were 2.5-4x more resistant (EC_50_ 169 nM), similar to other recent reports (Fig. 1A) [4, 8, 23]. Following expansion, CD8^+^ and CD4^+^ T cells became ~50x fold (EC_50_ 2,020 nM) and ~10x fold (EC_50_ 611 nM) more resistant to venetoclax respectively while Tregs had no change (Fig. 1a). However, Tregs expanded in the presence of BCL-2 blockade were 50x fold more resistant to subsequent venetoclax challenge (EC_50_ 7020 nM) followed by CD4^+^ T cells (4x fold, EC_50_ 2440 nM) and CD8^+^ T cells (1.4x fold, EC_50_ 2870 nM) (Fig. 1A). Venetoclax did not alter the overt “effector memory” phenotype of any expanded T cell subset (Fig. S1).

We next examined if the changes in sensitivity to venetoclax were reflective of differences in anti-apoptotic expression. As predicted, all T cell subsets significantly upregulated the main BCL-2 family regulators of T cell survival, BCL-2, BCL-X_L_, MCL-1, and BIM following expansion (Figs. 1B, C and S2) [7, 11, 24, 25]. Stimulation and IL-2 are known to increase expression of BCL-2 and BCL-X_L_ [26, 27]. Interestingly however, venetoclax treatment led to an even greater upregulation of anti-apoptotic proteins (Figs. 1B, D and S2). BCL-2 family mRNA expression changes following expansion were initially greater in CD8^+^ and CD4^+^ T compared to Tregs, but venetoclax blunted this while promoting increased expression in Tregs (Fig. S3). The relative levels of *Bcl-2, Bcl-xl, Mcl-1, Bim, Bax, and Bak* in all T cells and treatments underscored their importance in T cell apoptotic regulation (Fig. S3C–E) [10, 23, 28–30].

We next sought to determine if the presence of venetoclax during expansion induced different anti-apoptotic dependencies in T cells. CD8^+^ T cells expanded in the presence of venetoclax, still presumably bound to BCL-2, continued to rely on BCL-2 while CD4^+^ T cell and Treg mitochondrial depolarization increased in response to NOXA and HRK BH3 domains, reflecting increased reliance on MCL-1 and BCL-X_L_ respectively (Fig. 1E). Overall, these results suggest that, at least ex vivo, Tregs and CD4^+^ T cells are less dependent on BCL-2 at baseline and are more able to alter their anti-apoptotic dependencies when BCL-2 is blocked compared to CD8^+^ T cells.

### CD8^+^ T cells are highly sensitive to venetoclax in vivo, while Tregs are relatively resistant

To determine if T cells have similar sensitivities under homeostatic conditions in vivo, mice were treated with increasing doses of venetoclax daily for 7 days. Treatment was universally well tolerated and resulted in a dose-dependent reduction in spleen and lymph node cellularity, white blood cells, lymphocytes, and reticulocytes, which was curious given that reticulocytes rely on BCL-X_L_ for survival (Figs. S4, S5) [31]. Thymocyte subpopulations were similar in all animals apart from decreased CD8^+^ (single positive; SP) and CD4^−^CD8^−^ double negative (DN1) thymocytes at the highest dose (Fig. S6). CD4^+^CD8^+^ double positive (DP) thymocytes were unchanged supporting their reliance upon BCL-X_L_ [32]. Although we focus on T cells, immunophenotypic analysis of other immune cells was also performed (Tables S1, S2). Of note, there appears to be greater sensitivity to venetoclax of T cells within the lymph nodes compared to those within the spleen or thymus. This is likely due to the increased proportion of naïve T cells within the lymph nodes but differences in venetoclax bioavailability cannot be ruled out.

**Fig. 2:**
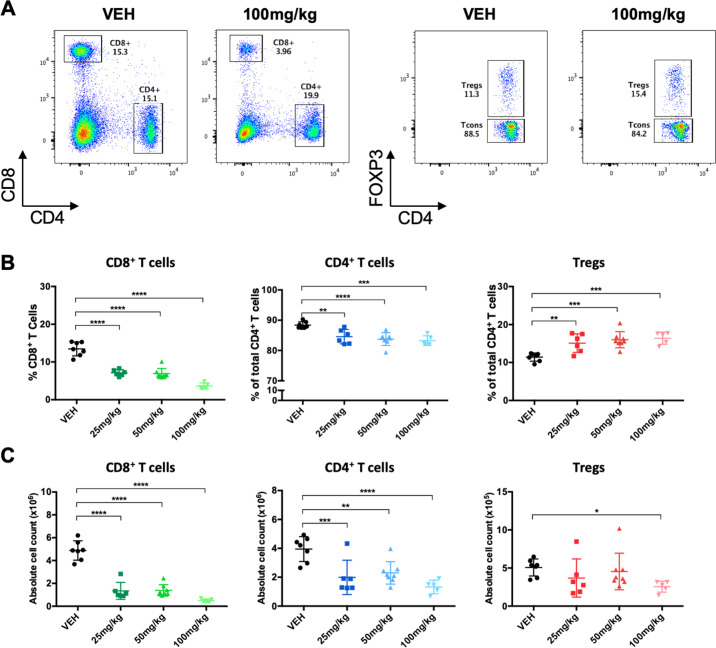
CD8^+^ T cells, CD4^+^ T cells, and Tregs have distinctive sensitivity patterns to short-term in vivo treatment with venetoclax. FOXP3-IRES-GFP mice were treated daily for 7 days with 25, 50, or 100 mg/kg venetoclax or vehicle control. **A** Representative flow plots of CD8^+^ T cells, CD4^+^ T cells and Tregs from the spleens of mice treated with 100 mg/kg venetoclax compared to vehicle. **B** Percentage and **C** Absolute number of CD8^+^ T cells, CD4^+^ T cells, and Tregs isolated from the spleens of treated animals. *n* ≥ 5 for each group of three independent experiments. Data represented as means ± SEM. *p < 0.05, **p < 0.01, ***p < 0.001, ****p < 0.0001.

**Fig. 3:**
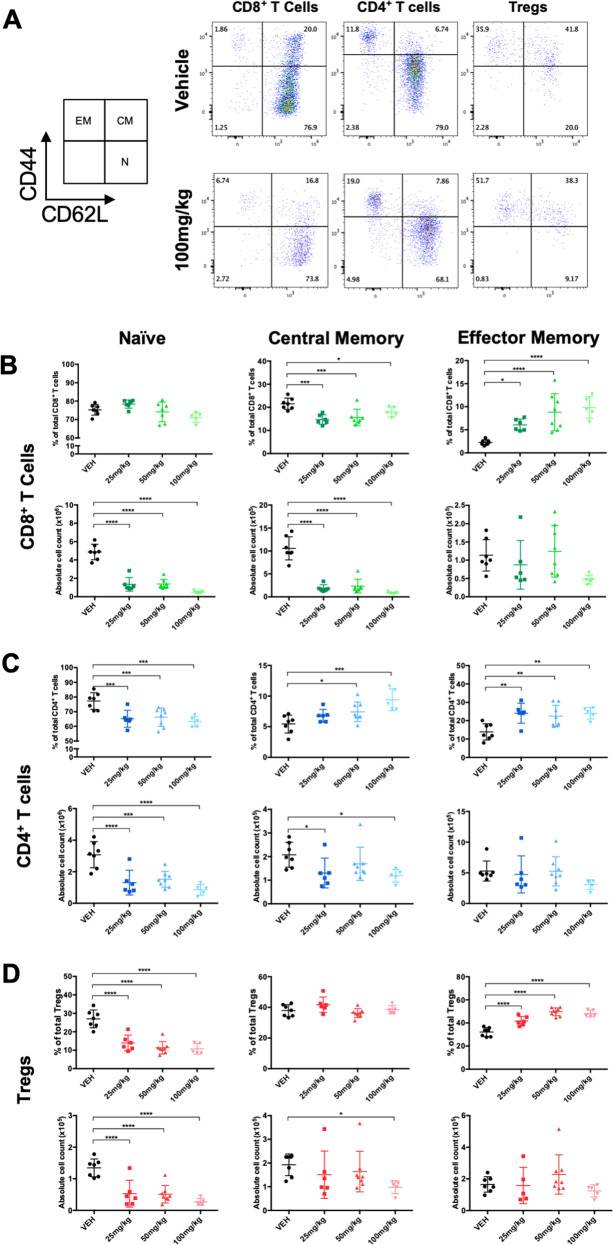
Short-term in vivo treatment with venetoclax leads to an enrichment of effector memory cells in all three T cell subsets. FOXP3-IRES-GFP mice were treated daily for 7 days with increasing doses of venetoclax or vehicle control. **A** Representative gating strategy for CD8^+^ T cells, CD4^+^ T cells, and Tregs from mice treated with 100 mg/kg venetoclax compared to vehicle. **B**–**D** Proportions of naive (CD44^low^CD62L^high^), effector memory (CD44^high^CD62L^low^), and central memory (CD44^high^CD62L^high^) T cells, shown as percentages (top) and absolute numbers (bottom). **B** CD8^+^ T cells (**C**) CD4^+^ T cells (**D**) Tregs. *n* ≥ 5 for each group. Data represented as means ± SEM of three independent experiments. *p < 0.05, **p < 0.01, ***p < 0.001, ****p < 0.0001.

As predicted, based on our ex vivo studies, venetoclax led to significant dose-dependent decreases in the percentage and absolute number of CD8^+^ and CD4^+^ T cells while the percentage of Tregs increased (Fig. 2). One limitation to measuring changes in T cells ex vivo is the necessity to expand cells, leading to a universal effector memory-like phenotype [33–35]. To better assess T cell sensitivity and adaptivity to venetoclax in surviving cells in vivo, we examined T cell subpopulations (Fig. 3). All naïve (CD44^low^CD62L^high^) and CD4^+^ and CD8^+^ central memory (CD44^high^CD62L^high^) T cells were particularly sensitive to venetoclax indicating reliance on BCL-2 (Fig. 3B–D). As a result, effector memory (CD44^high^CD62L^low^) T cell numbers remained relatively stable and their relative percentages increased. While the majority of CD4^+^ and CD8^+^ T cells are naïve at steady state, Tregs were more equally distributed into naïve, central memory, and effector memory phenotypes (Fig. 3D). Thus, short-term venetoclax treatment in vivo causes significant changes in the global T cell landscape with relative sparing of all memory Tregs, which was likely partly responsible for their overall lower baseline sensitivity to venetoclax ex vivo (Fig. 1).

**Fig. 4:**
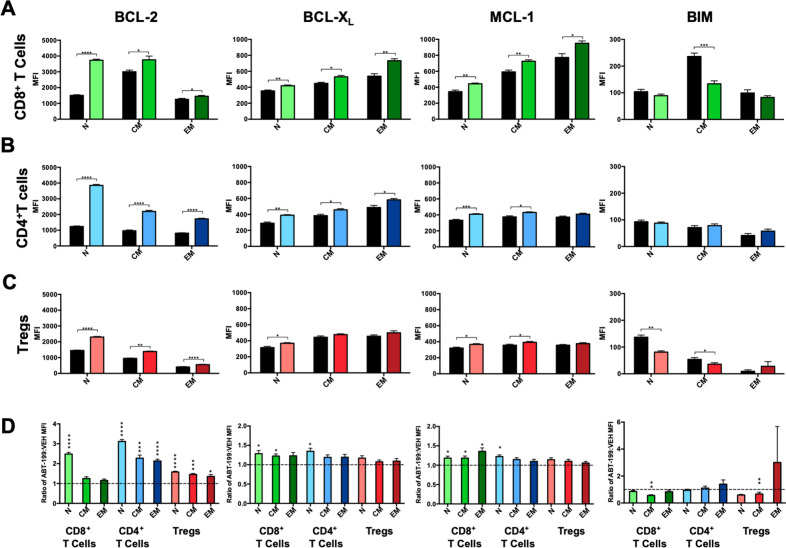
Short-term in vivo treatment with venetoclax leads to an increase in anti-apoptotic protein levels, especially of BCL-2, in all three T cell subsets. FOXP3-IRES-GFP mice were treated daily for 7 days with 50 mg/kg venetoclax or vehicle control. Protein levels of key BCL-2 family members were measured in naïve, effector memory, and central memory subsets of **A** CD8^+^ T cells, **B** CD4^+^ T cells, and **C** Tregs. Data is shown as mean fluorescence intensity. **D** Ratios of protein levels in T cells from venetoclax-treated animals compared to vehicle-treated controls. *n* = 4 mice for each group. Data represented as means ± SEM of three independent experiments. *p < 0.05, **p < 0.01, ***p < 0.001, ****p < 0.0001.

We next wanted to determine if the remaining T cells following treatment adapted to BCL-2 blockade similarly as when activated ex vivo (Fig. 1). While BCL-2 was significantly increased in all remaining T cells, particularly in naïve cells, BCL-X_L_ and MCL-1 levels also rose, especially in CD8^+^ and CD4^+^ T cells (Fig. 4). BIM levels either remained the same or decreased (Fig. 4). These results indicate that short-term venetoclax treatment alters the homeostatic T cell landscape and differentially alters anti-apoptotic levels in T cells.

### T cells adapt to BCL-2 blockade through development to maintain T cell homeostasis

**Fig. 5:**
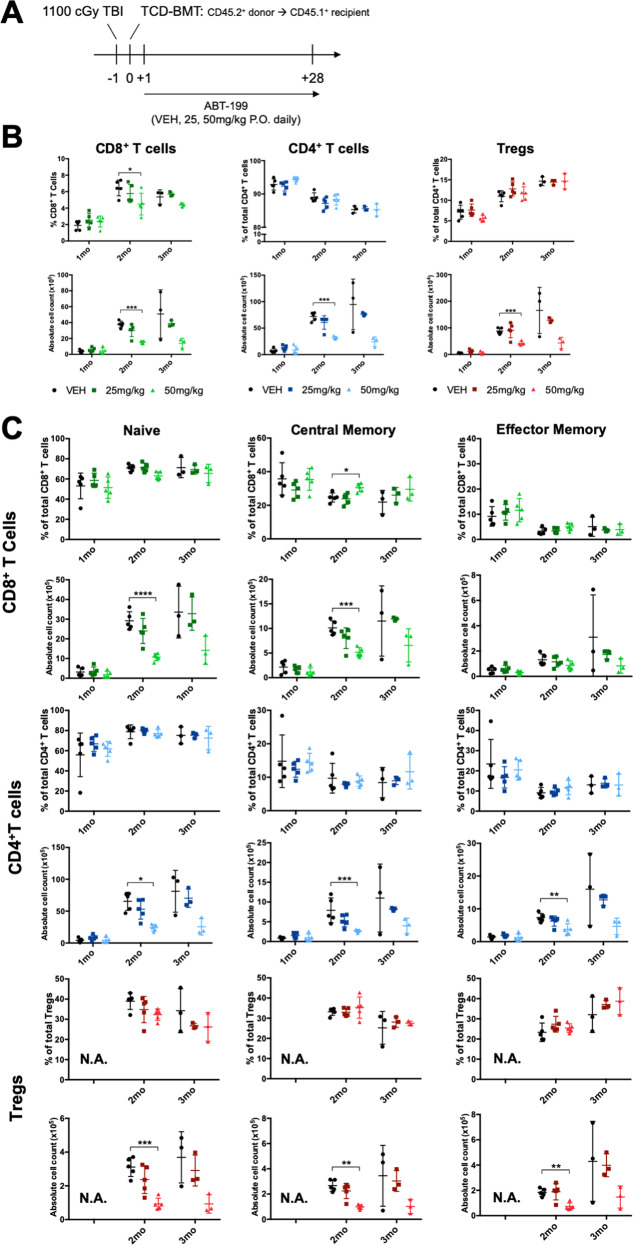
Long-term in vivo treatment with venetoclax following bone marrow transplantation does not affect the relative proportions of any T cell subset. **A** Schematic of transplant procedure where recipient CD45.1^+^ mice were lethally irradiated on day -1 and were transplanted with 2 × 10^6^T cell-depleted (TCD) bone marrow from FOXP3-IRES-GFP (CD45.2^+^) donors on day 0. Daily oral gavage treatment with 25 or 50 mg/kg venetoclax was initiated on day +1 and continued until day +28 post-transplant. **B** Percentage (top) and absolute numbers (bottom) of CD8^+^ T cells, CD4^+^ T cells, and Tregs, shown as subsets of total CD4^+^ T cells isolated from animals 1, 2, and 3 months post-transplant. **C** Proportions of naive (CD44^low^CD62L^high^), effector memory (CD44^high^CD62L^low^), and central memory (CD44^high^CD62L^high^) T cells, shown as percentages (top) and absolute numbers (bottom). N.A. = the low number of Tregs one-month post-transplant precluded their thorough analysis at this time. *n* = 5 mice/group for the 1 and 2 month timepoints and *n* = 3 for the 3 month timepoint. Data represented as means ± SEM of two independent experiments. *p < 0.05, **p < 0.01, ***p < 0.001, ****p < 0.0001.

**Fig. 6:**
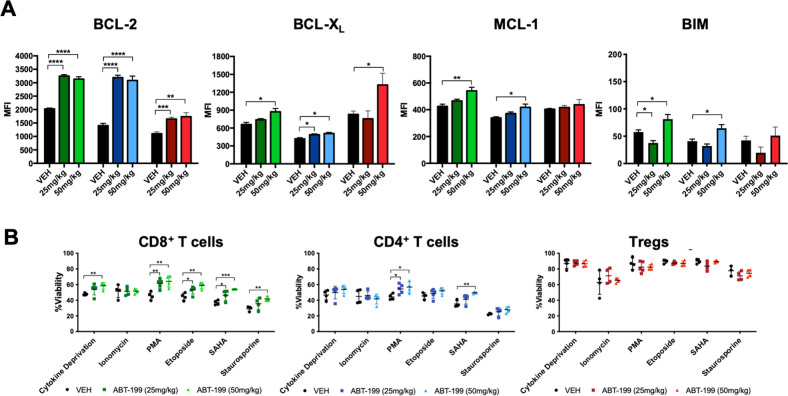
Long-term in vivo treatment with venetoclax following bone marrow transplantation results in similar increases in BCL-2 levels but different cell death sensitivity between T cell subtypes. **A** Protein levels of naïve CD8^+^ T cells, CD4^+^ T cells and Tregs one-month post-transplant. *n* ≥ 3 mice/group. **B** CD8^+^ T cells, CD4^+^ T cells, and Tregs from mice at the 2 month timepoint were treated with apoptotic stimuli for 24 h. Viability of T cells was assessed by Annexin V/PI staining with Annexin V^neg^PI^neg^ cells considered viable. *n* ≥ 4 mice/group. Data represented as means ± SEM of two independent experiments. *p < 0.05, **p < 0.01, ***p < 0.001, ****p < 0.0001.

We next tested if longer BCL-2 blockade would lead to similar phenotypic skewing and anti-apoptotic reprogramming in T cells akin to short-term treatment. To evaluate T cells that have been equally treated, rather than a mix of variously aged cells, we utilized an autologous transplant model in which CD45.1^+^ mice were transplanted with T cell-depleted bone marrow from FOXP3-IRES-GFP (CD45.2^+^) mice and given vehicle, 25 mg/kg, or 50 mg/kg of venetoclax daily through day +28 post-transplant (Fig. 5A). Animals tolerated venetoclax but developed a slight dose-dependent decrease in splenocytes, lymphocytes, and thymocytes extending 3 months post-transplant (Fig. S7). Despite these changes, mice showed no significant differences in the proportions of donor-derived CD8^+^, CD4^+^, or Treg cells immediately following treatment suggesting T cell adaptivity (Figs. 5B, S8) [36]. However, there was a dose-dependent reduction in T cell numbers 2 and 3 months post-transplant, through the period of normal post-transplant homeostatic expansion (Fig. 5B) [37]. Despite this, the relative percentages of naïve, central memory, and effector memory T cells were near normal immediately following treatment and beyond (Fig. 5C). Similar to animals treated short-term, venetoclax failed to induce major changes in thymocyte populations. However, treatment resulted in significant upregulation of BCL-2, and to a lesser extent BCL-X_L_ and MCL-1, in certain thymocytes (Fig. S9) [32]. These changes extended to mature T cells in treated animals (Fig. 6A). BIM expression was relatively stable in all T cells but was downregulated slightly in animals treated with 25 mg/kg and upregulated in those treated with 50 mg/kg indicating perhaps a biphasic, dose-dependent, response akin to what was measured following ex vivo expansion (Fig. 1). Despite increased levels of anti-apoptotic proteins and blocked BCL-2, venetoclax did not result in abnormal skewing of TCRVβ repertoires (Fig. S10). CD8^+^ T cells from venetoclax-treated animals were significantly resistant to a wider range of BCL-2 family-mediated cell death stimuli compared to CD4^+^ T cells (Fig. 6B). Tregs were equally resistant to all stimuli whether or not they matured in the presence of venetoclax (Fig. 6B) [38]. The relative proportions of CD4^+^, CD8^+^, and Treg naive (CD44^low^) and memory (CD44^high^) cells in each treatment condition were not significantly different, similar to what was measured in Fig. 5 (Fig. S11). Thereby, the cell death differences measured were not the result from selective depletion of naïve or memory cells. In conclusion, proportions of T cell subsets are unchanged secondary to long-term BCL-2 blockade post-transplant. However, such treatment induced significant long-lasting changes in BCL-2 family protein levels and cell death sensitivities, reflecting inherent differences between T cells in their ability to acclimate to long-term BCL-2 blockade.

### Venetoclax leads to transcriptional alterations consistent with stimulated cells

**Fig. 7:**
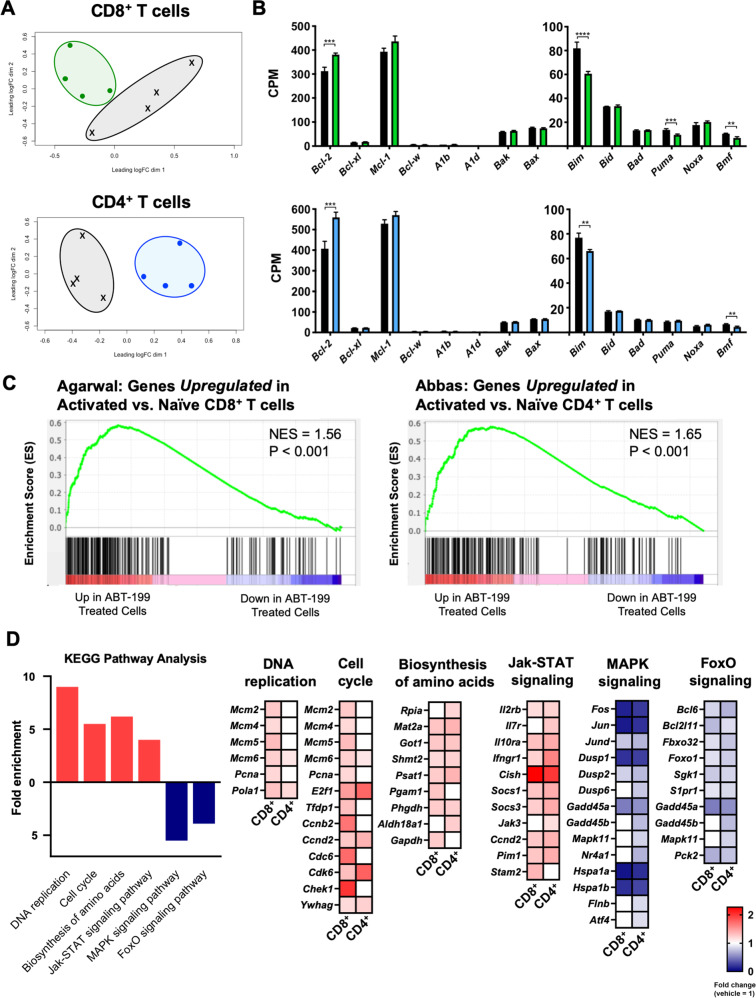
Long-term BCL-2 blockade leads to changes in BCL-2 family transcripts and upregulation of genes consistent with an activated phenotype. Mice were treated as described in Fig. 5 and naïve CD8^+^ and CD4^+^ T cells were collected for RNA-sequencing one day following the last dose of venetoclax. **A** Multidimensional principle component analysis of gene expression from CD8^+^ and CD4^+^ isolated from venetoclax-treated (green and blue areas respectively) compared to vehicle-treated controls (black areas). **B** Counts per million reads (CPM) of the BCL-2 family genes, separated by multidomain anti-/pro-apoptotic and BH3-only proteins. **C** Gene set enrichment data of RNA-sequencing data performed using the ‘Immunological Signatures’ collection in the Molecular Signatures Database. Gene sets used were: GSE15930 for CD8^+^ T cell analysis [68] and GSE22886 for CD4^+^ T cell analysis [3]. **D** Transcriptional profiles of CD8^+^ and CD4^+^ T cells by KEGG pathway analysis of significantly upregulated (red) and downregulated (blue) genes (*p* < 0.01). For all pathways enriched, individual genes are displayed in heatmap arrays according to fold change in expression normalized to vehicle with additional comparison between CD8^+^ and CD4^+^ T cells within each panel. *n* = 4 mice/group. *p < 0.05, **p < 0.01, ***p < 0.001, ****p < 0.0001.

To determine if venetoclax led to changes beyond BCL-2 family protein levels we performed RNA sequencing on naïve T cells immediately following treatment post-transplant. Indeed, venetoclax led to distinct transcriptional profiles for CD8^+^ and CD4^+^ T cells as demonstrated by multidimensional principle component analysis (Fig. 7A). RNA-sequencing identified 143 and 501 differentially expressed genes with a significance cutoff of 0.01 FDR in CD8^+^ and CD4^+^ T cells respectively (Fig. S12A, B). 23% of differentially expressed genes were shared by CD8^+^ and CD4^+^ T cells. Transcript levels of the two most important BCL-2 family regulators of T cell survival, *Bcl-2* and *Bim*, were significantly increased and decreased respectively in both T cell subsets (Fig. 7B). However, the most significantly shared upregulated genes were those associated with T cell activation, including *Irf4*, *Irf8*, *Batf*, *Cish*, and *Cd69* (Fig. S12C). Gene set enrichment analysis further determined that genes normally expressed in stimulated/activated T cells were positively enriched in venetoclax-treated cells (Fig. 7C). Further analysis indicated similar altered pathways between CD4^+^ and CD8^+^ T cells suggesting a shared mechanism of action (Fig. 7D). There was most significant upregulation in genes associated with cell division and JAK/STAT signaling and downregulation in genes associated with MAPK and FoxO signaling. Thus, long-term BCL-2 blockade of T cells post-transplant during expansion not only changed their apoptotic rheostat, but also altered their global gene expression profiles to reflect an activated-like state.

## Discussion

**Fig. 8:**
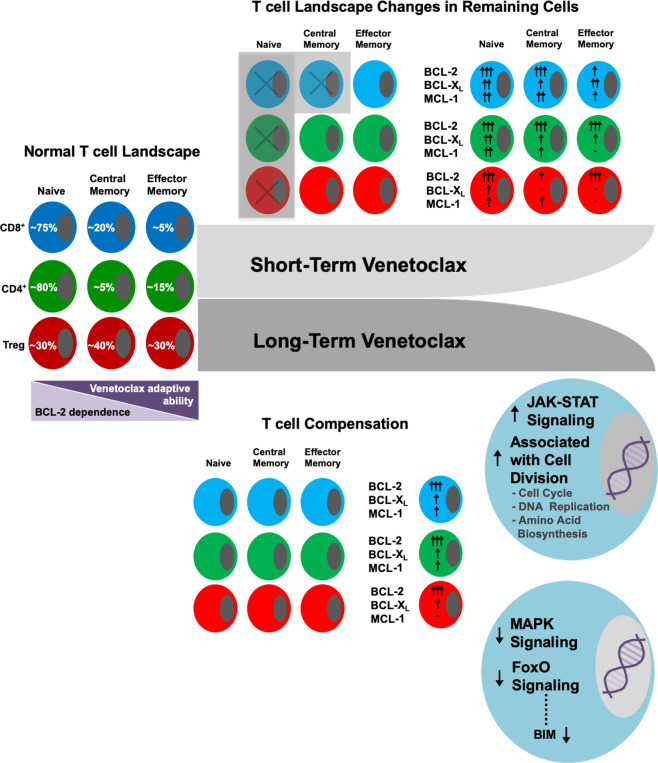
Summary of differences in effects of short-term versus long-term venetoclax treatment in T cells. Normal percentages of naïve, central memory, and effector memory CD8^+^, CD4^+^, and Treg cells along with their respective cell death dependence on BCL-2 and “adaptive” ability to alter anti-apoptotic dependency patterns in the setting of BCL-2 blockade is shown on the left. Short-term treatment with venetoclax alters the T cell landscape predominantly through killing most naïve CD8^+^, CD4^+^, and Treg cells and central memory CD8^+^ T cells. Surviving cells significantly upregulate BCL-2 protein expression. Surviving Tregs, on whole, do not alter their anti-apoptotic protein levels as much as CD8^+^ and CD4^+^ T cells. Long-term treatment with venetoclax, however, does not alter the relative percentages of T cell subtypes but does result in significantly increased BCL-2 protein expression in all T cells indicating BCL-2 family and T cell survival adaptation to BCL-2 blockade. Additionally, T cells treated long-term have significant changes in their mRNA expression patterns reflecting a more activated signature.

It is well known that T cells have a shifting reliance on BCL-2 proteins during development, activation, contraction, and maintenance [10, 28, 39]. Much of what is known has been determined primarily through gene deletion animal models, while much less is understood on how T cells respond to anti-apoptotic targeting using BH3 mimetics. Our current study supports recent evidence that naïve T cells are sensitive to short-term treatment with venetoclax [4, 5, 40], and is the first to show that BCL-2 targeting in CD8^+^, CD4^+^, and Tregs in vitro and in vivo significantly and, rather uniformly, increase BCL-2, BCL-X_L_, and MCL-1 protein levels making these cells more resistant to cell death (Fig. 8). Because anti-apoptotic proteins in aggregate, and not alone, determine cell death sensitivity, our results point to differences between the ability of surviving T cells to acclimate or adapt to BCL-2 inhibition with venetocolax [41]. Interestingly, venetoclax-induced protein level changes were not solely due to increased transcription, suggesting that competition for and removal of endogenous BH3-only proteins bound to BCL-2, such as BIM, may stabilize anti-apoptotic proteins within T cells, similar to what has been observed for certain peptide and small molecule-based BH3-mimetics in cancerous cells [42–44]. However, the exact mechanism(s) behind this are unknown and are currently being studied.

Overall, Tregs and CD4^+^ T cells were more effective at adapting to BCL-2 inhibition compared to CD8^+^ T cells both ex vivo and during short-term in vivo treatment (Fig. 8). The comparatively lower BCL-2 upregulation in Tregs was perhaps one reason why Tregs were not as affected by BCL-2 targeting in vivo. Whether this is due to Tregs’ lower dependency on BCL-2, greater dependency on MCL-1 at baseline, or that Tregs are able to more effectively modulate BCL-2 protein family dependence against venetoclax is unclear [40, 45]. Effector memory cells were resistant to venetoclax in all three T cell subsets, consistent with reports examining effector cells treated with ABT-737 and supporting that other anti-apoptotic proteins are more pertinent for T cell survival during the transition from a naïve to a memory state, such as MCL-1, BCL-X_L_, and A1 [46–52]. Despite large changes in T cell subsets in the periphery, thymocytes were only minimally affected by venetoclax treatment. Although BCL-2 is expressed at higher levels in DN and CD4 and CD8 SP cells, these cells are likely dependent on additional anti-apoptotic proteins such as MCL-1 for their survival in this setting [25, 53].

Clinically however, patients are not given short bursts of venetoclax. Rather, they are treated with either single-agent venetoclax daily or following chemotherapy for up to 28 days as the marrow repopulates and the immune system homeostatically expands [54–59]. There are no studies of which we are aware that investigate how such treatment with venetoclax affects T cell development. In this setting, although we found no reduction in T cell proportions following treatment, there was longer-lasting lower cell numbers. While CD8^+^ T cells that matured in the presence of venetoclax had no survival disadvantage compared to other T cells, naïve CD8^+^ T cells and CD4^+^ T cells had increased levels of BCL-2 and were resistant to a range of apoptotic stimuli, indicating apoptotic adaptation. Again, the immediate and longer-lasting BCL-2-associated changes between CD8^+^ and CD4^+^ T cells suggest that they uniquely adapt, akin to differences we previously measured in T cells lacking *Bim* [11]. These results emphasize that the context in which the BCL-2 family of proteins are targeted may greatly affect T cell death sensitivity, similar to what has been measured in BCL-2 family genetic deletion models [10, 11]. How these changes relate to immune function in patients is currently unknown. Intriguingly, recent reports of two clinical trials where venetoclax was given for 21 and 28 days at 400 mg and 800 mg daily respectively found increased infections/sepsis in patients who received venetoclax, yet there were no differences in lymphopenia between those patients receiving placebo or the higher dose venetoclax [54, 60]. One speculation was that venetoclax-induced unknown functional effects on lymphocytes [60].

Changes in T cells treated with venetoclax through development were not limited to the BCL-2 family. There was shared significant downregulation of *Jun* subfamilies (c*-Jun, JunB, JunD*) and c-*Fos*, genes which encode proteins critical for T cell function through their interaction with NFAT to bind AP-1 regulatory elements [61–63]. AP-1 (Fos/Jun) activity is controlled in large part by MAP kinases and genes within the MAPK signaling pathway, which were significantly downregulated in treated naïve CD4^+^ and CD8^+^ T cells. How these genetic changes are related to BCL-2 inhibition and affect T cell function or metabolism is unclear and an area we are currently investigating. Additionally, whether venetoclax induces genetic “adaptation” versus particular T cell apoptotic selection (*i.e*. naïve T cell killing) followed by expansion of this specific T cell subgroup is currently unknown. This along with downregulation of FoxO and upregulation of Jak-STAT signaling may be responsible for the recently reported increased efficacy of tumor specific CD8^+^ effector T cells in mice treated with venetoclax daily for 14 days and even more robustly in combination with anti-PD-1/PD-L1 blockade [4]. Verma et al. [64] also found that inhibition of the MAPK signaling pathway through MEK1/2 inhibition induces strong anti-tumor activity and reprogramming of effector CD8^+^ T cells. Additionally, FoxO target genes are involved in cell cycle and apoptotic control and directly regulate the expression of *Bim* [65, 66]. Downregulation of this pathway may have been responsible for the decrease in *Bim* expression in venetoclax-treated cells and may be related to the upregulation of genes associated with cell cycle, DNA replication, and amino acid biosynthesis. It is unclear why the larger (50 mg/kg) dose of venetoclax decreased *Bim* mRNA expression but resulted in slightly increased BIM protein in bulk CD8^+^ and CD4^+^ T cells post-transplant. While the majority of these cells were naïve, it is possible that memory cells played a role in this increase. In conclusion, while there is evidence that genetic changes occur in direct response to venetoclax treatment in cancer cells, our study is the first to show that venetoclax also imparts genetic reprogramming in normal T cells [67]. The durability of these changes is an area of active investigation.

Our results highlight the dynamism and adaptive capabilities of T cells in response to BCL-2 inhibition and how such treatment, either for short or long periods of time, reshapes the immune system. Future studies are aimed at determining the mechanism responsible for the genetic changes found in T cells following BCL-2-blockade and how these affect immune functionality in various scenarios. We also believe that further exploration is warranted into how venetoclax and other BH3-mimetics may affect other immune cells, particularly in the context of anti-cancer therapy, for which these agents are currently being studied and used clinically.

## Supplementary information

Supplemental Data

Fig S1

Fig S2

Fig S3

Fig S4

Fig S5

Fig S6

Fig S7

Fig S8

Fig S9

Fig S10

Fig S11

Fig S12

Table S1

Table S2

## Data Availability

All RNA-sequencing data are uploaded to the NCBI GEO database under the accession number GSE164483.
