# Supplementary material for: Venetoclax imparts distinct cell death sensitivity and adaptivity patterns in T cells
Source: Cell Death Dis. 2021 Oct 27;12(11):1005. doi: 10.1038/s41419-021-04285-4 (PMC8551340; doi:10.1038/s41419-021-04285-4)
Supplement: Supplementary file 1 — Supplemental Data [file 41419_2021_4285_MOESM1_ESM.docx]

Venetoclax imparts distinct cell death sensitivity and adaptivity patterns in T cells

Lindsey M. Ludwig^1^, Katrina M. Hawley^1^, David B. Banks^1,2^, Anika T. Thomas-Toth^1^, Bruce R. Blazar ^3^, Megan E. McNerney ^1,4^, Joel D. Leverson^5^, James L. LaBelle^1^

^1^ Department of Pediatrics, Section of Hematology/Oncology, University of Chicago, Chicago, IL.

^2^ Medical Scientist Training Program, University of Chicago, Chicago, IL

^3^ Department of Pediatrics, Division of Blood and Marrow Transplantation, University of Minnesota, Minneapolis, MN.

^4^ Department of Pathology, University of Chicago, Chicago, IL.

^5^ AbbVie Inc., North Chicago, IL.

Address Correspondence to:

James L. LaBelle

900 East 57^th^ Street, KCBD 5122, Chicago, IL 60637

Email: [jlabelle@peds.bsd.uchicago.edu](mailto:jlabelle@peds.bsd.uchicago.edu)

Phone: 773-702-6812

Fax: 773-834-1329

Running Title**:** Venetoclax treatment alters T cell landscape

**Supplemental Data:**

**Fig. S1: CD8^+^ T cells, CD4^+^ T cells, and Tregs have a similar effector memory-like (CD44^high^, CD62L^low^) phenotype following stimulation and expansion in the absence or presence of venetoclax.** Representative flow cytometric analysis of treated and untreated CD8^+^ T cells, CD4^+^ T cells and Tregs after expansion. The relative expression of CD25 is equally similar in all T cells subtypes in tested.

**Fig. S2: Intracellular flow of BCL-2, BCL-X_L_, MCL-1, and BIM in *ex vivo* stimulated T cells in the presence of absence of venetoclax.** Colored data (CD8^+^ T cells in green, CD4^+^ T cells in blue, and Tregs in red) represent treatment following stimulation (dark green, blue, and red) or following stimulation in the presence of venetoclax (light green, blue, and red). Data is representative of 3 independent replicates.

**Fig. S3: CD8^+^ T cells, CD4^+^ T cells, and Tregs vary in their BCL-2 family expression following stimulation and expansion in the presence of venetoclax.** **A** BCL-2 family mRNA expression levels of stimulated CD8^+^ T cells (green) , CD4^+^ T cells (blue) and Tregs (red). **B** BCL-2 family mRNA expression levels of expanded CD8^+^ T cells, CD4^+^ T cells and Tregs in the presence of venetoclax. Unstimulated T cells were used as reference samples for both **A** and **B**. C-E. Cycle threshold (C_T_) values for individual BCL-2 family protein mRNA for CD8^+^ T cells, CD4^+^ T cells and Tregs at rest (**C**), following stimulation/expansion alone (**D**) or in the presence of venetoclax (**E**). Expression for parts (**A)** and (**B)** was normalized using UBC and analyzed using the ∆∆C_T_ method. Gene Expression Probes were as follows: Mcl-1:Mm00725832 s1; Bak1:Mm00432045 m1; Bcl2l11/Bim:Mm00437796 m1; Bcl2a1a/Bfl-1/A1:Mm03646861 mH; Bbc3/Puma:Mm00519268 m1; Pmaip1/Noxa:Mm00451763 m1; Bad:Mm00432042 m1; Bid:Mm00477631 m1; Bcl-2:Mm00477631 m1; Bcl2l1/Bcl-xL:Mm00437783 m1; Bcl2l2/Bcl w):Mm03053297 s1; Bax:Mm00432051 m1; Bmf:Mm00506773 m1; UBC:Mm02525934_g1.

**Fig. S4: Short-term treatment with venetoclax is well tolerated but causes significantly decreased cell counts in the spleen and lymph nodes.** FOXP3-IRES-GFP mice were treated with increasing doses of venetoclax orally daily for 7 days. **A** Mouse body weight. **B** Spleen weight and total splenocyte count. **C** Total cell counts from the lymph nodes and (**D**) thymi. n≥5 for each group. Data represented as means ±SEM. *p<0.05, ** p<0.01, *** p<0.001, **** p<0.0001.

**Fig. S5: Effects on constituents of peripheral blood in animals treated short-term with venetoclax.** FOXP3-IRES-GFP mice were treated with increasing doses of venetoclax administered orally daily for 7 days. Complete blood counts were measured from peripheral blood and analyzed for (**A**) absolute total white blood cell (WBC) content, (**B**) hemoglobin, (**C**) red blood cells (RBC), (**D**) platelets, (**E**) absolute neutrophil count and percentage, (**F**) absolute lymphocyte count and percentage, (**G**) absolute monocyte count and percentage, (**H**) absolute eosinophil count and percentage, (**I**) absolute basophile count and percentage, and (**J**) absolute reticulocyte count and percentage . n≥3 for each group. Data represented as means ±SEM. *p<0.05, ** p<0.01, *** p<0.001, **** p<0.0001.

**Fig. S6: Short-term treatment with venetoclax does not cause overt phenotypic changes to developing thymocytes. A** Representative flow cytometric plots of thymocytes from each treatment group. **B** Percentages of CD4^+^ SP, CD8^+^ SP, CD4^+^CD8^+^ DP, and CD4^−^CD8^−^ DN thymocytes. **C** Percentages of DN subsets: DN1 (CD44^+^CD25^−^), DN2 (CD44^+^CD25^+^), DN3 (CD44^−^CD25^+^), and DN4 (CD44^−^CD25^−^). n≥5 for each group. Data represented as means ±SEM. *p<0.05, ** p<0.01, *** p<0.001, **** p<0.0001.

**Fig. S7: Long-term daily treatment with venetoclax is well tolerated following autologous bone marrow transplantation with TBI conditioning.** CD45.1^+^ recipient mice were myeloablated and transplanted with autologous T cell-depleted CD45.2^+^ bone marrow. Mice were treated with 25mg/kg or 50mg/kg venetoclax daily for 28 days following transplantation. **A** Mouse body weights during venetoclax treatment and at 1, 2, and 3 months post-transplant. **B** Absolute cell counts from the spleen, lymph nodes, and thymi of animals 1, 2, and 3 months post-transplant. n=5 mice/group for the 1 and 2 month timepoints and n=3 for the 3 month timepoint. Data represented as means ±SEM. *p<0.05, ** p<0.01, *** p<0.001, **** p<0.0001.

**Fig. S8: Daily venetoclax treatment does not affect donor T and B cell engraftment following autologous bone marrow transplantation. A** Proportions of donor (CD45.2^+^) and host (CD45.1^+^) lymphocytes from the spleen, lymph nodes, and thymi of transplanted mice. **B** Proportions of donor-derived B220^+^ B cells, CD4^+^ T cells, and CD8^+^ T cells following transplant. n=5 mice/group for the 1 and 2 month timepoints and n=3 for the 3 month timepoint. Data represented as means ±SEM. *p<0.05, ** p<0.01, *** p<0.001, **** p<0.0001.

**Fig. S9: Venetoclax treatment does not affect overall thymocyte phenotype post-transplant but does increase expression of anti-apoptotic BCL-2 proteins in individual T cell populations. A** Representative flow plots of thymi from each treatment group at one-month post-transplant. **B** Percentages of CD4^+^ SP, CD8^+^ SP, CD4^+^CD8^+^ DP, and CD4^−^CD8^−^ DN thymocytes. **C** Percentages of DN subsets: DN1 (CD44^+^CD25^−^), DN2 (CD44^+^CD25^+^), DN3 (CD44^−^CD25^+^), and DN4 (CD44^−^CD25^−^). **D** Protein levels of CD4^+^ SP, CD8^+^ SP, CD4^+^CD8^+^ DP, and CD4^−^CD8^−^ DN thymocytes 1-month post-transplant. Data is shown as MFI of each antibody subtracted from the IgG isotype control. n=5 mice/group. Data represented as means ±SEM. *p<0.05, ** p<0.01, *** p<0.001, **** p<0.0001.

**Fig. S10: Long-term treatment with venetoclax does not skew the TCR V**β **repertoire of CD8^+^ T cells, CD4^+^ T cells, or Tregs.** Percentages of TCR Vβ chain usage 2 months following transplantation, 1 month after venetoclax treatment, of (**A)** CD8^+^ T cells, (**B)** CD4^+^ T cells and (**C**) Tregs. Characterization of the TCR Vβ repertoire was performed using the TCR Vβ screening panel (BD). Data represented as means ±SEM. *p<0.05, ** p<0.01, *** p<0.001, **** p<0.0001.

**Fig. S11: Long-term treatment with venetoclax does not significantly alter the relative percentages of naïve and memory T cells.** Percentages of naïve (CD44^low^) and memory (CD44^high^) in (**A)** CD8^+^ T cells, (**B)** CD4^+^ T cells and (**C**) Tregs used in the viability assays shown in Figure 6B. n≥4 mice/group. Data represented as means ±SEM.

**Fig. S12: Differentially expressed genes in naïve CD8^+^ and CD4^+^ T cells isolated from transplanted animals following venetoclax treatment. A** Overlay of the number of shared and unshared genes that were upregulated and downregulated in venetoclax treated versus vehicle-treated CD8^+^ (green) and CD4^+^ (blue) T cells. 64 and 236 genes were significantly upregulated in CD8^+^ and CD4^+^ T cells respectively, while 79 and 265 were significantly downregulated. **B** Volcano plots of differentially expressed genes in CD8^+^ and CD4^+^ T cells relative to vehicle controls. The significance cutoff is p≤0.01 and the fold change cutoff is 1.0 (red points). **C** CPM of genes related to T cell activation state in CD8^+^ T cells and CD4^+^ T cells. n=4 mice/group. *p<0.05, ** p<0.01, *** p<0.001, **** p<0.0001.

**Table S1: Absolute numbers of immune cell subpopulations in the spleen, lymph nodes, and thymi of animals treated with various doses of venetoclax for seven days.** Antibodies used for phenotyping were: FOXP3-GFP, CD25-APC(PC61), CD4-APC(RM4-5) or CD4-PE(RM4-5) and CD8-FITC(53-6.7), CD4-PE-Cy7(RM4-5), CD4-APC(RM4-5), CD8-PE(53-6.7), CD8-BUV395(53-6.7), CD62L-PE(MEL-14), CD25-BUV39(PC61), CD44-APC(IM7), CD44-PE-Cy7(IM7), CD8-FITC(53-6.7), CD45.2-BUV737(104), CD45.1-APC(A20), IgM-PE(DS-1), B220-APC(RA3-6B2), B220-BUV395(RA3-6B2), CD3-PE-Cy7(17A2), γδ-TCR-PE(GLZ), β-chain TCR-APC(H57-597), NK1.1-BUV395(PK136), CD43-APC(S7), IgD-BUV39(11-26c.2a), CD23-APC(B3B4), and CD21/35-PE-Cy7(7E9). All antibodies were purchased from BD biosciences (BD), Biolegend, Cell Signaling Technology, or eBioscience.

**Table S2: Mean percentages of immune cell subpopulations in the spleen, lymph nodes, bone marrow, and thymi of animal treated with various doses of venetoclax for seven days.** Antibodies used for phenotyping were: FOXP3-GFP, CD25-APC(PC61), CD4-APC(RM4-5) or CD4-PE(RM4-5) and CD8-FITC(53-6.7), CD4-PE-Cy7(RM4-5), CD4-APC(RM4-5), CD8-PE(53-6.7), CD8-BUV395(53-6.7), CD62L-PE(MEL-14), CD25-BUV39(PC61), CD44-APC(IM7), CD44-PE-Cy7(IM7), CD8-FITC(53-6.7), CD45.2-BUV737(104), CD45.1-APC(A20), IgM-PE(DS-1), B220-APC(RA3-6B2), B220-BUV395(RA3-6B2), CD3-PE-Cy7(17A2), γδ-TCR-PE(GLZ), β-chain TCR-APC(H57-597), NK1.1-BUV395(PK136), CD43-APC(S7), IgD-BUV39(11-26c.2a), CD23-APC(B3B4), and CD21/35-PE-Cy7(7E9). All antibodies were purchased from BD biosciences (BD), Biolegend, Cell Signaling Technology, or eBioscience.
