# Supplementary figures and images for: Venetoclax imparts distinct cell death sensitivity and adaptivity patterns in T cells
Source: Cell Death Dis. 2021 Oct 27;12(11):1005. doi: 10.1038/s41419-021-04285-4 (PMC8551340; doi:10.1038/s41419-021-04285-4)

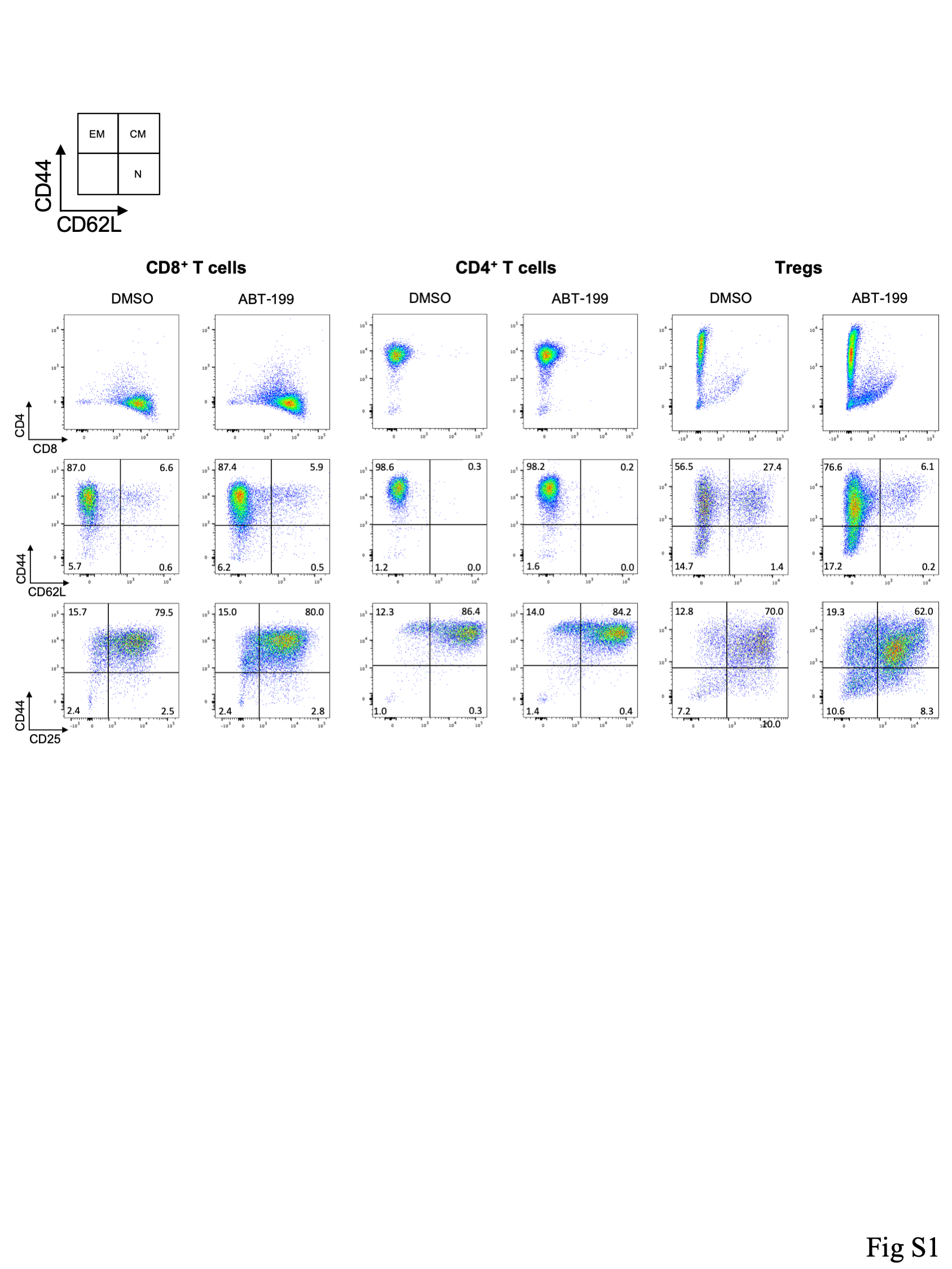

Supplement: Supplementary file 2 — Fig S1 [file 41419_2021_4285_MOESM2_ESM.tif]

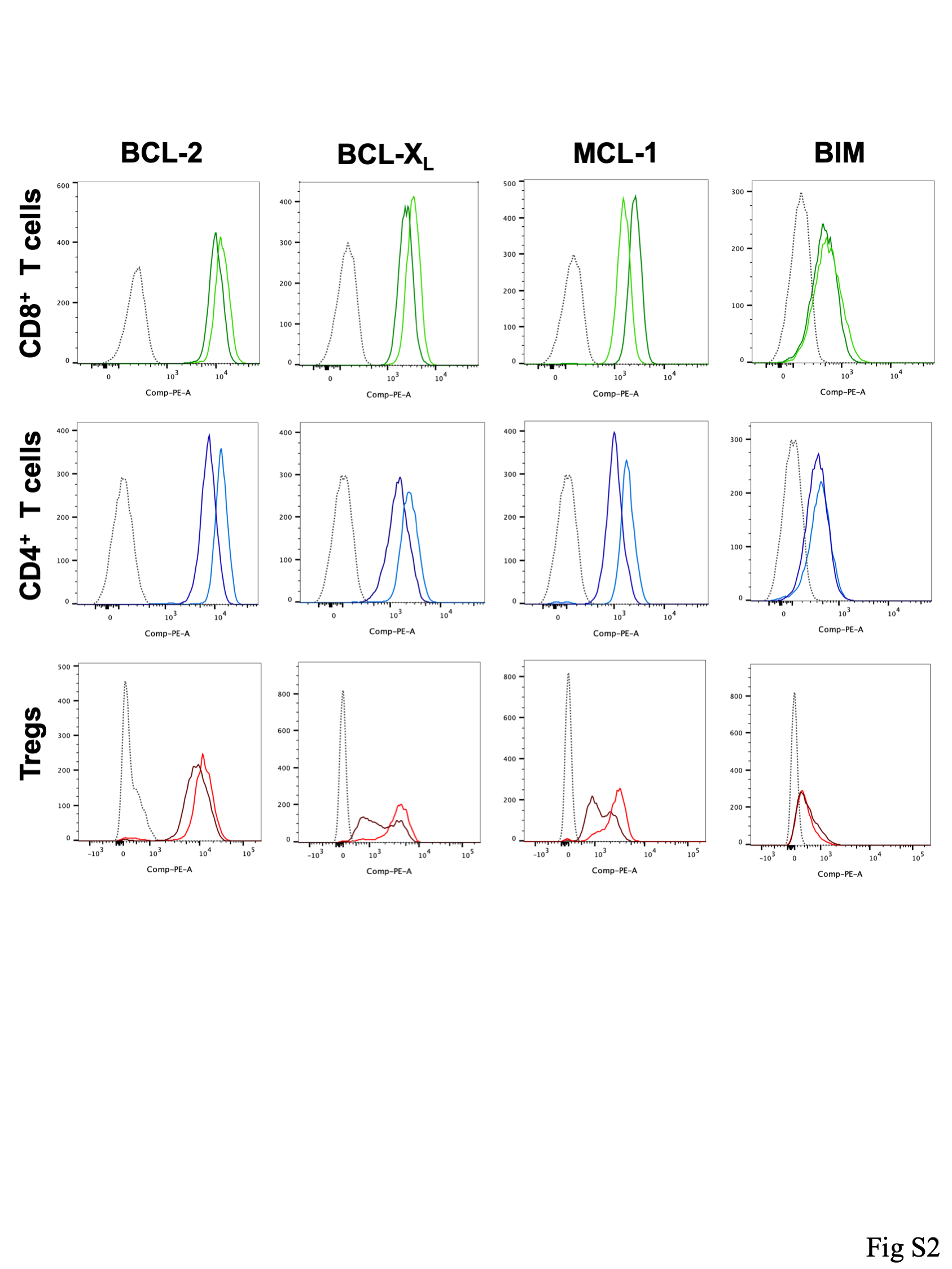

Supplement: Supplementary file 3 — Fig S2 [file 41419_2021_4285_MOESM3_ESM.tif]

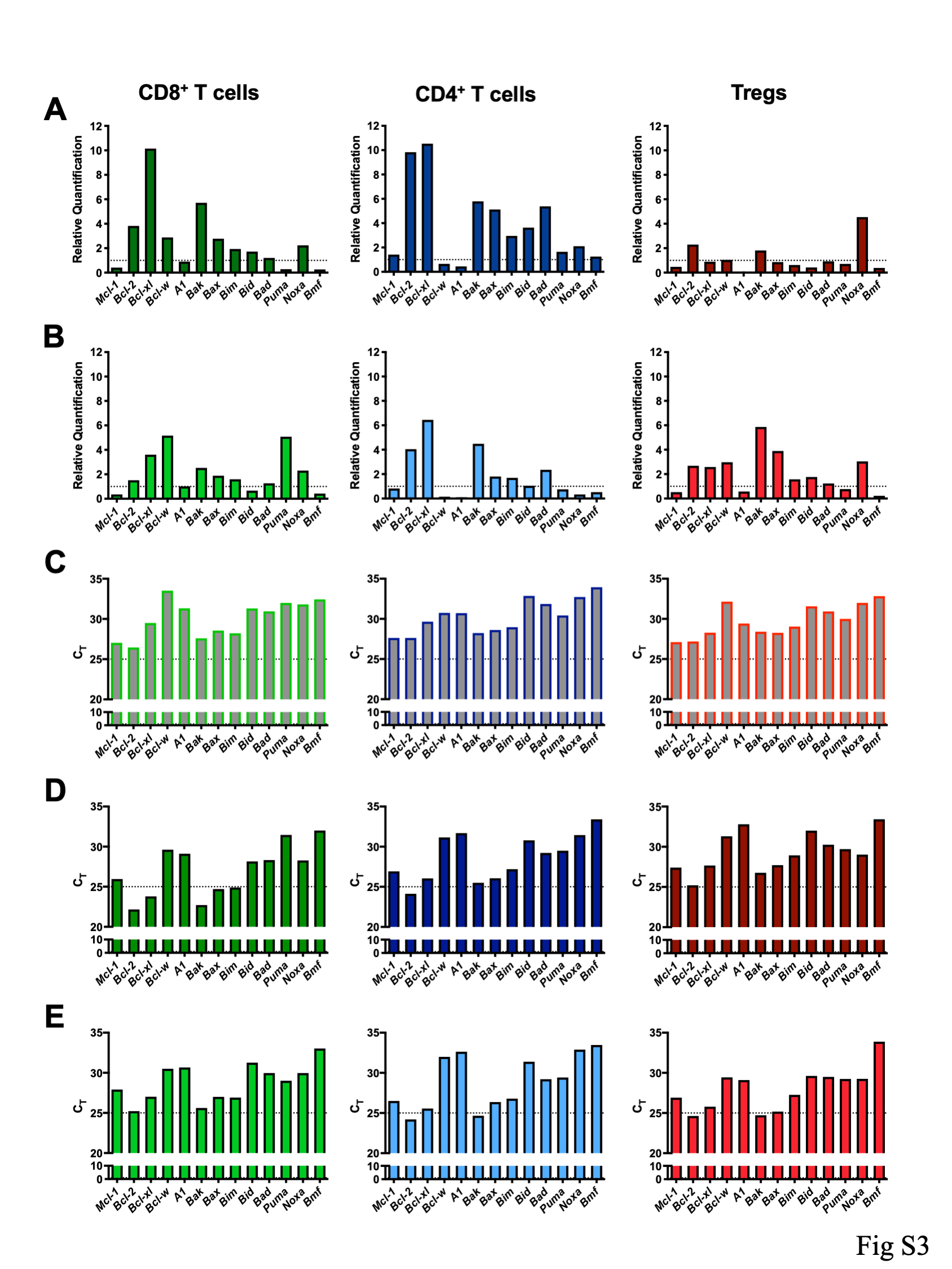

Supplement: Supplementary file 4 — Fig S3 [file 41419_2021_4285_MOESM4_ESM.tif]

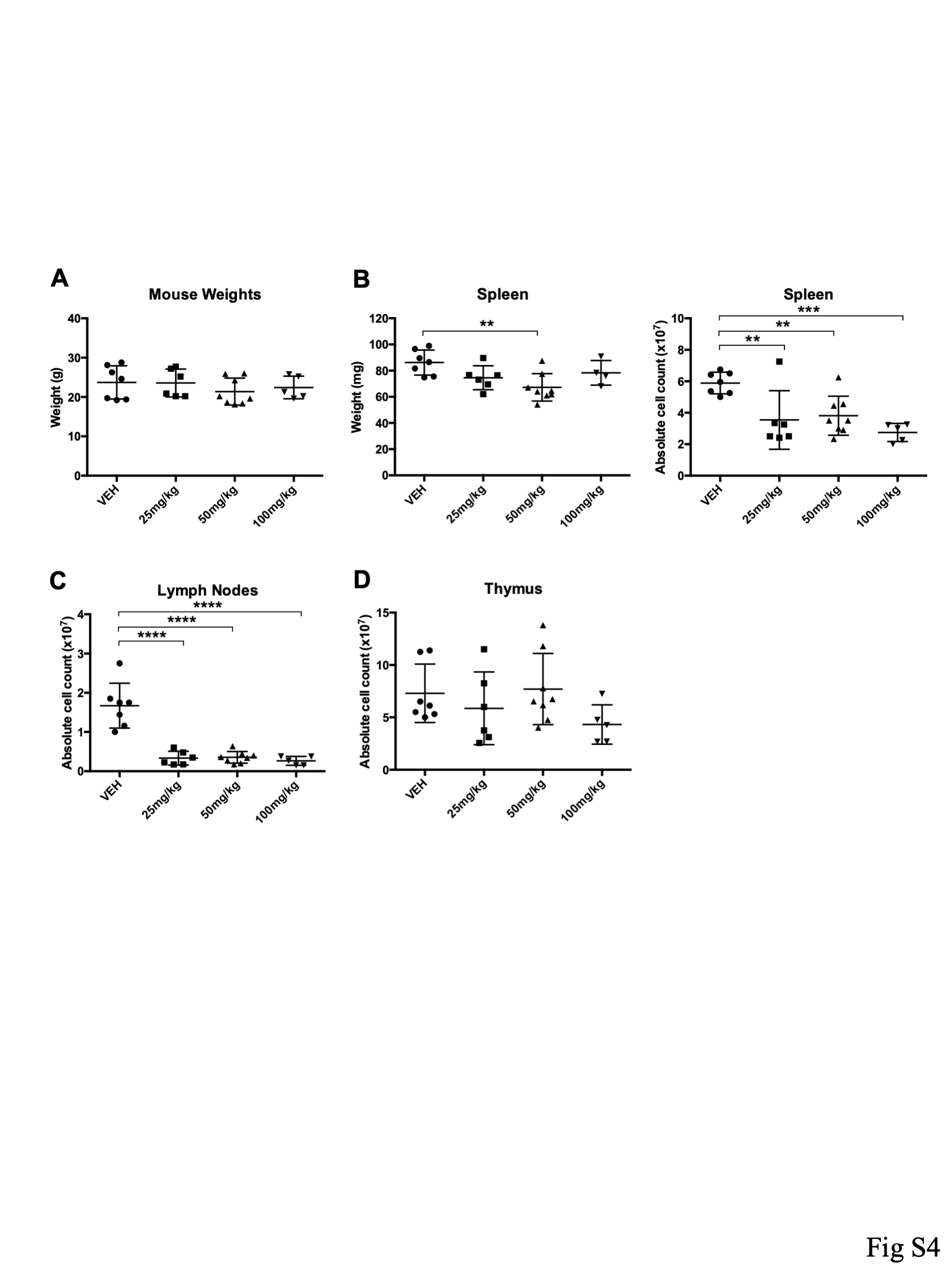

Supplement: Supplementary file 5 — Fig S4 [file 41419_2021_4285_MOESM5_ESM.tif]

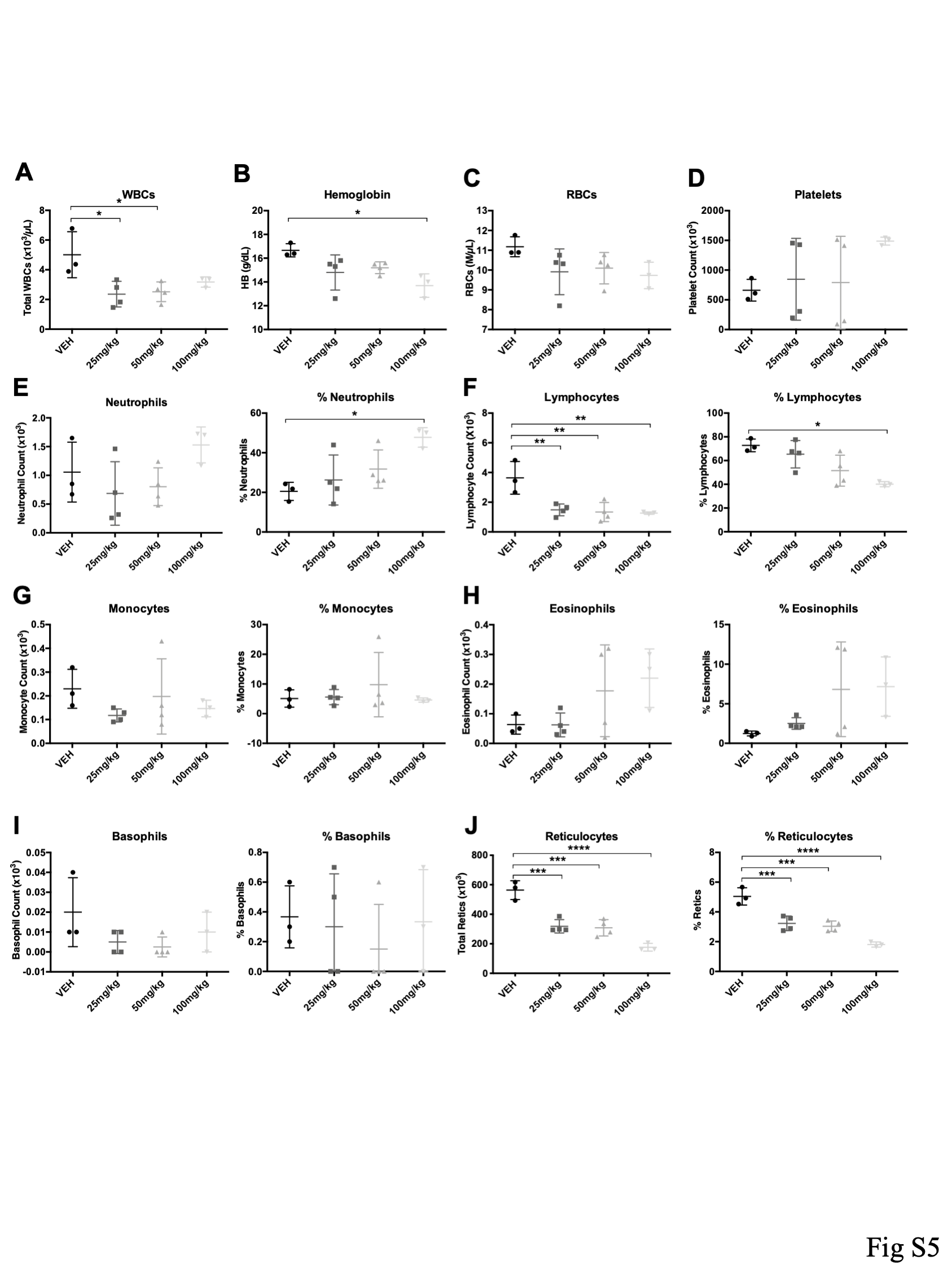

Supplement: Supplementary file 6 — Fig S5 [file 41419_2021_4285_MOESM6_ESM.tif]

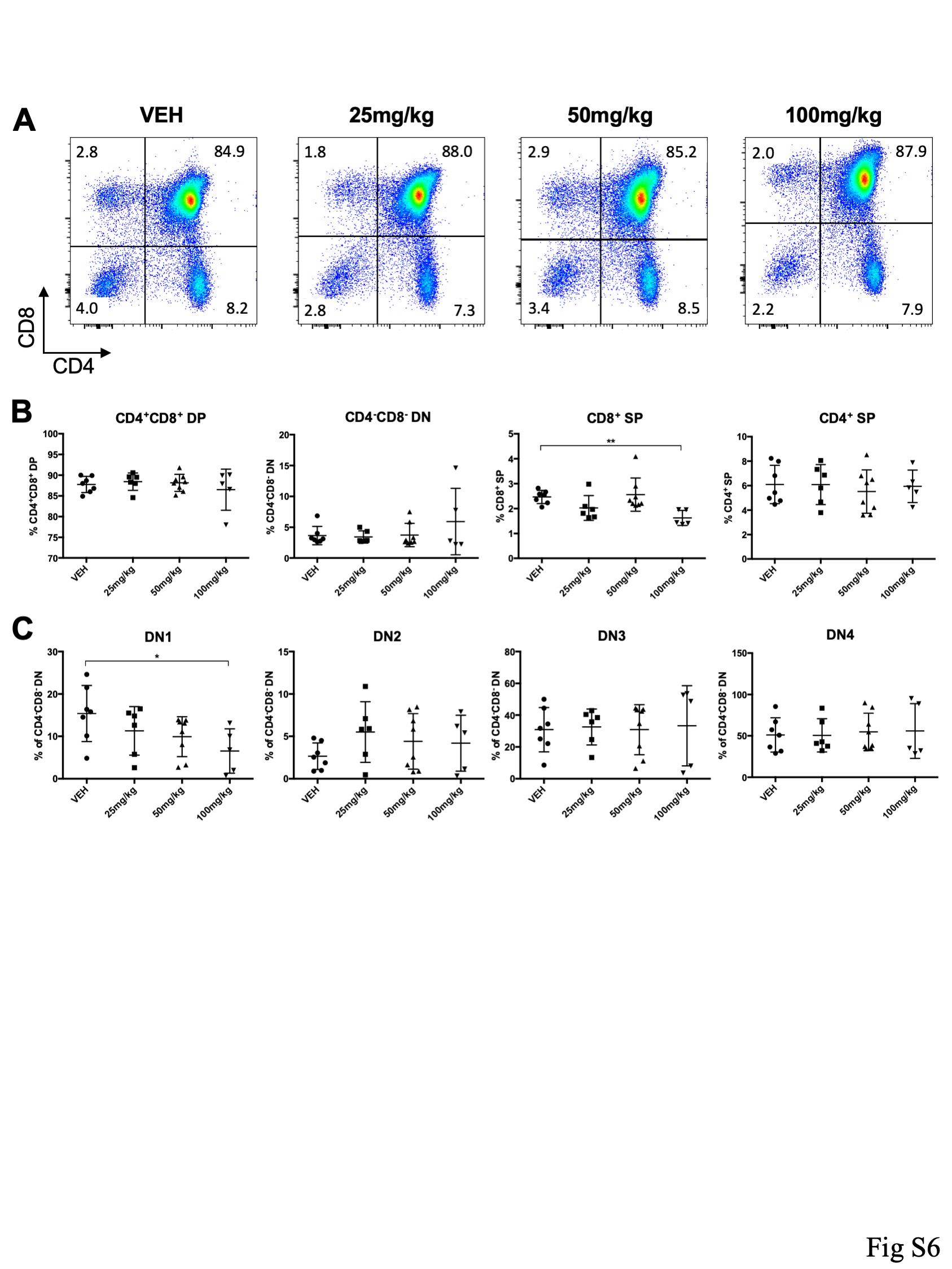

Supplement: Supplementary file 7 — Fig S6 [file 41419_2021_4285_MOESM7_ESM.tif]

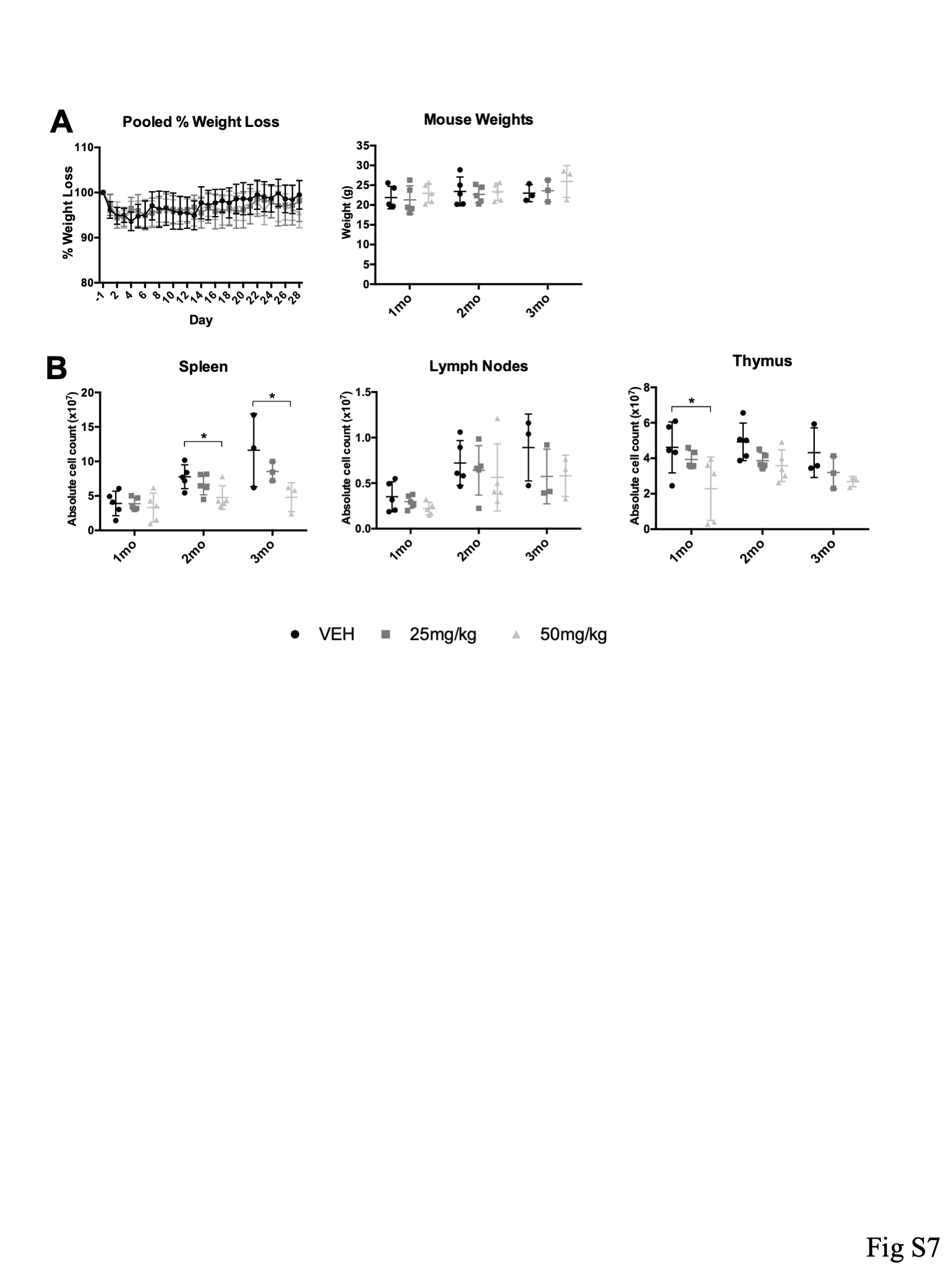

Supplement: Supplementary file 8 — Fig S7 [file 41419_2021_4285_MOESM8_ESM.tif]

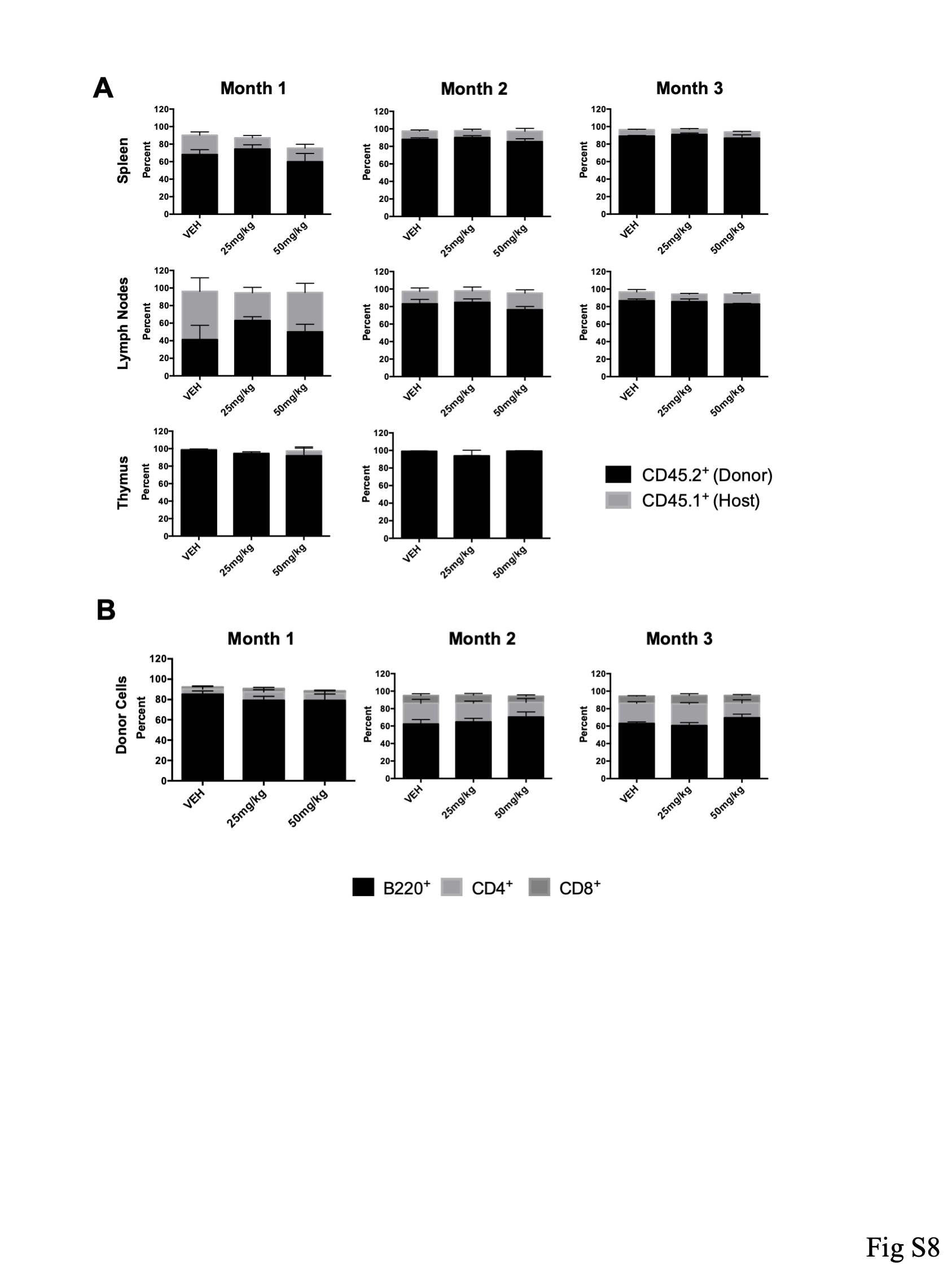

Supplement: Supplementary file 9 — Fig S8 [file 41419_2021_4285_MOESM9_ESM.tif]

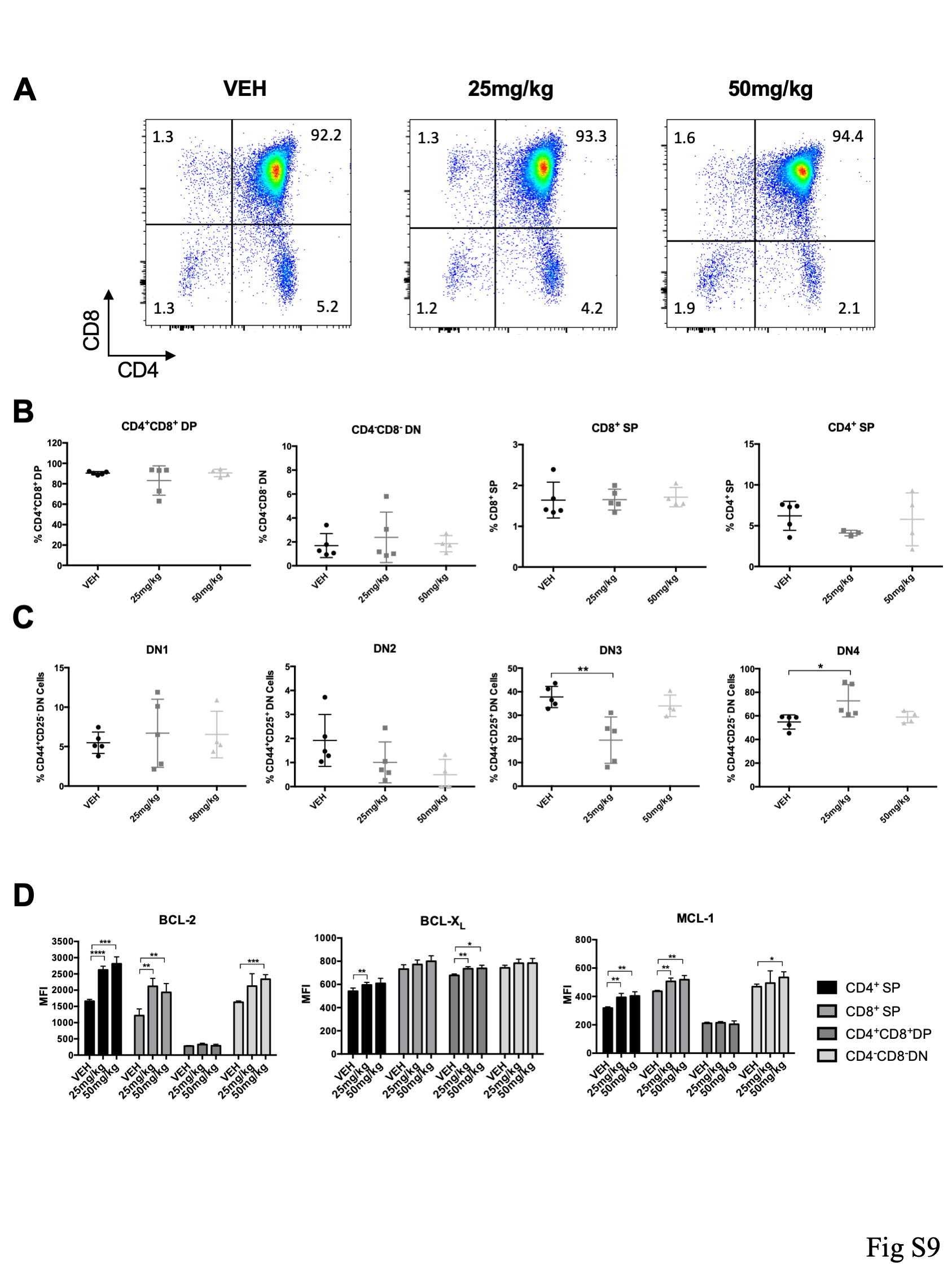

Supplement: Supplementary file 10 — Fig S9 [file 41419_2021_4285_MOESM10_ESM.tif]

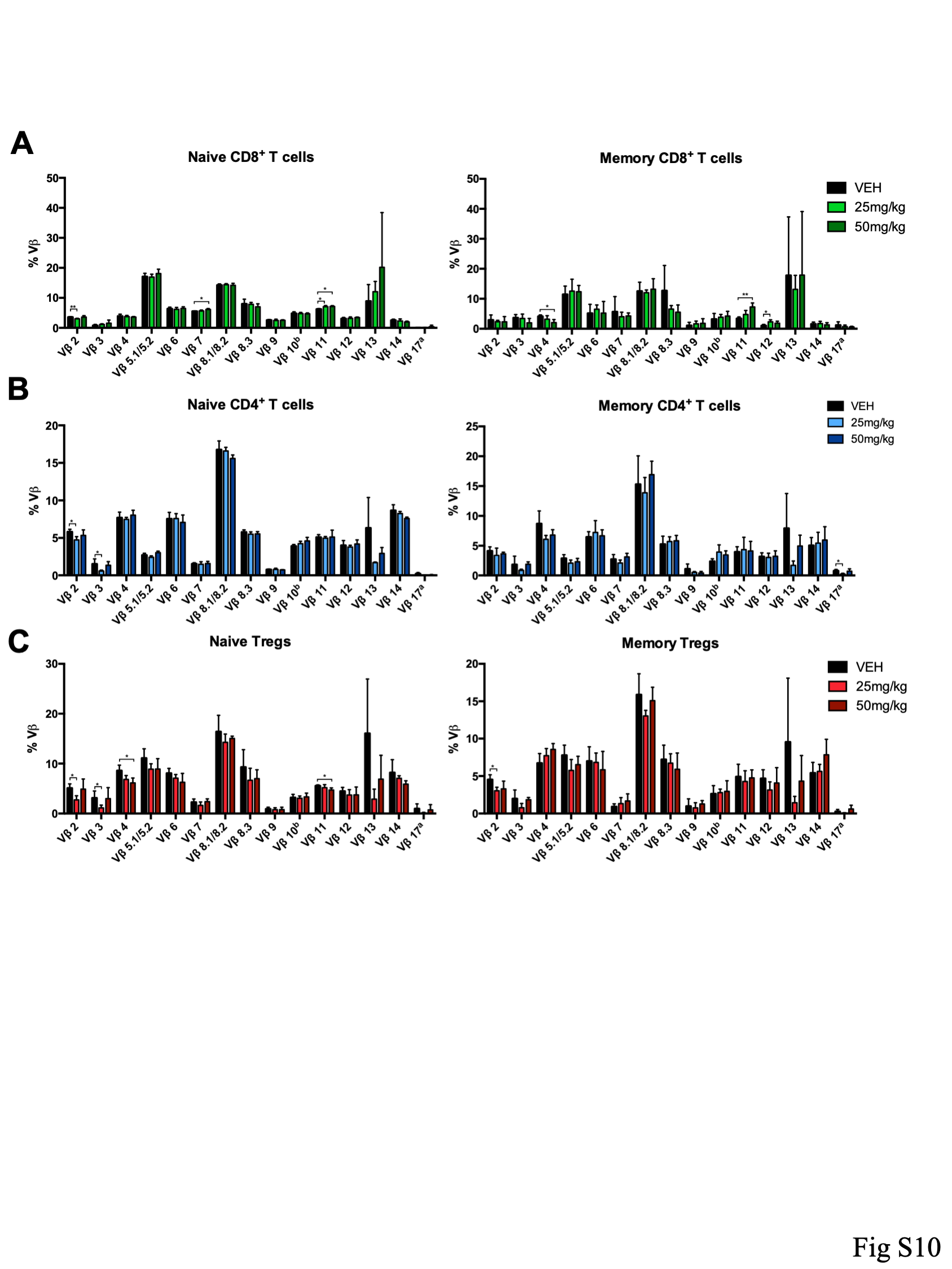

Supplement: Supplementary file 11 — Fig S10 [file 41419_2021_4285_MOESM11_ESM.tif]

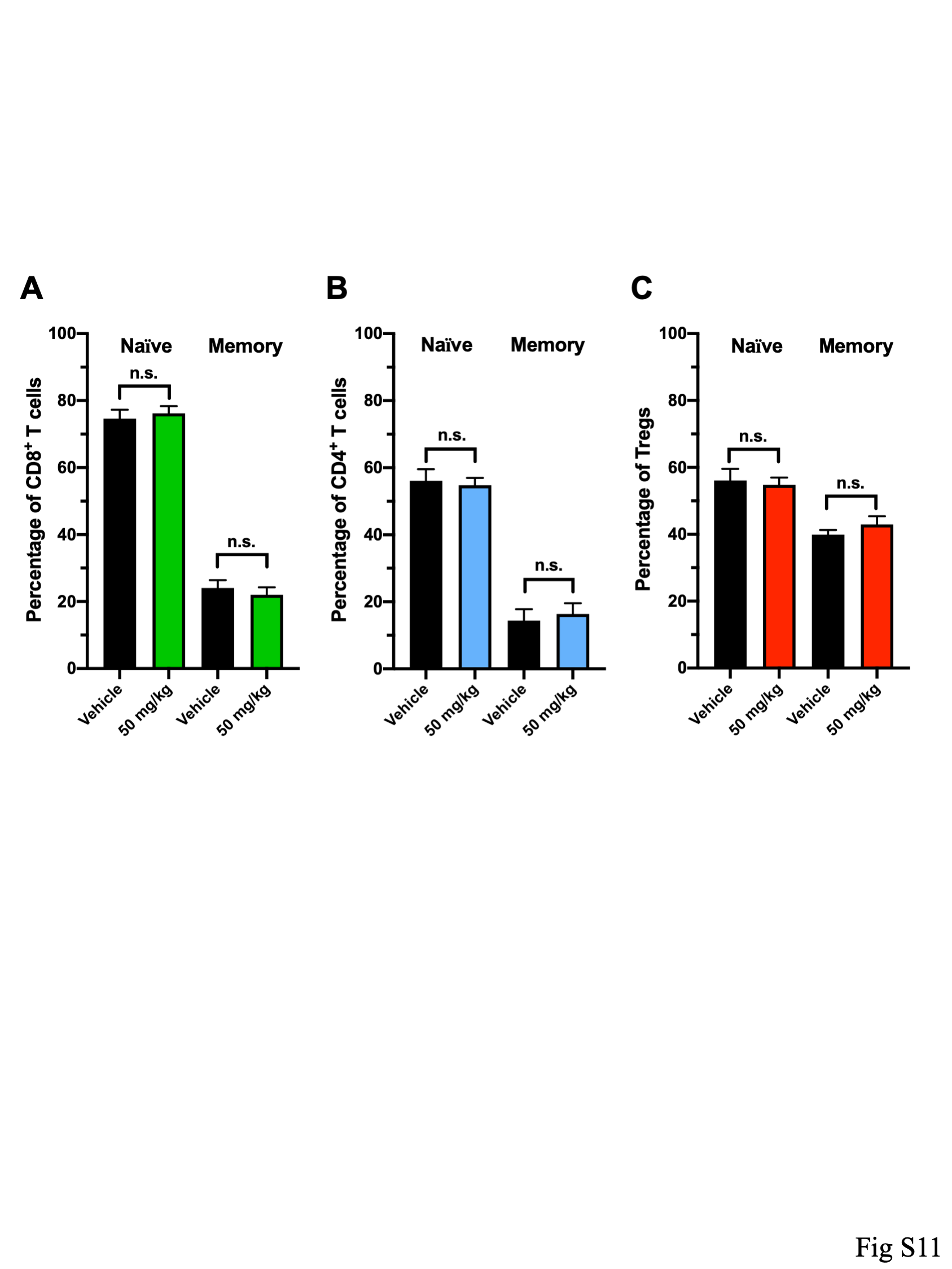

Supplement: Supplementary file 12 — Fig S11 [file 41419_2021_4285_MOESM12_ESM.tif]

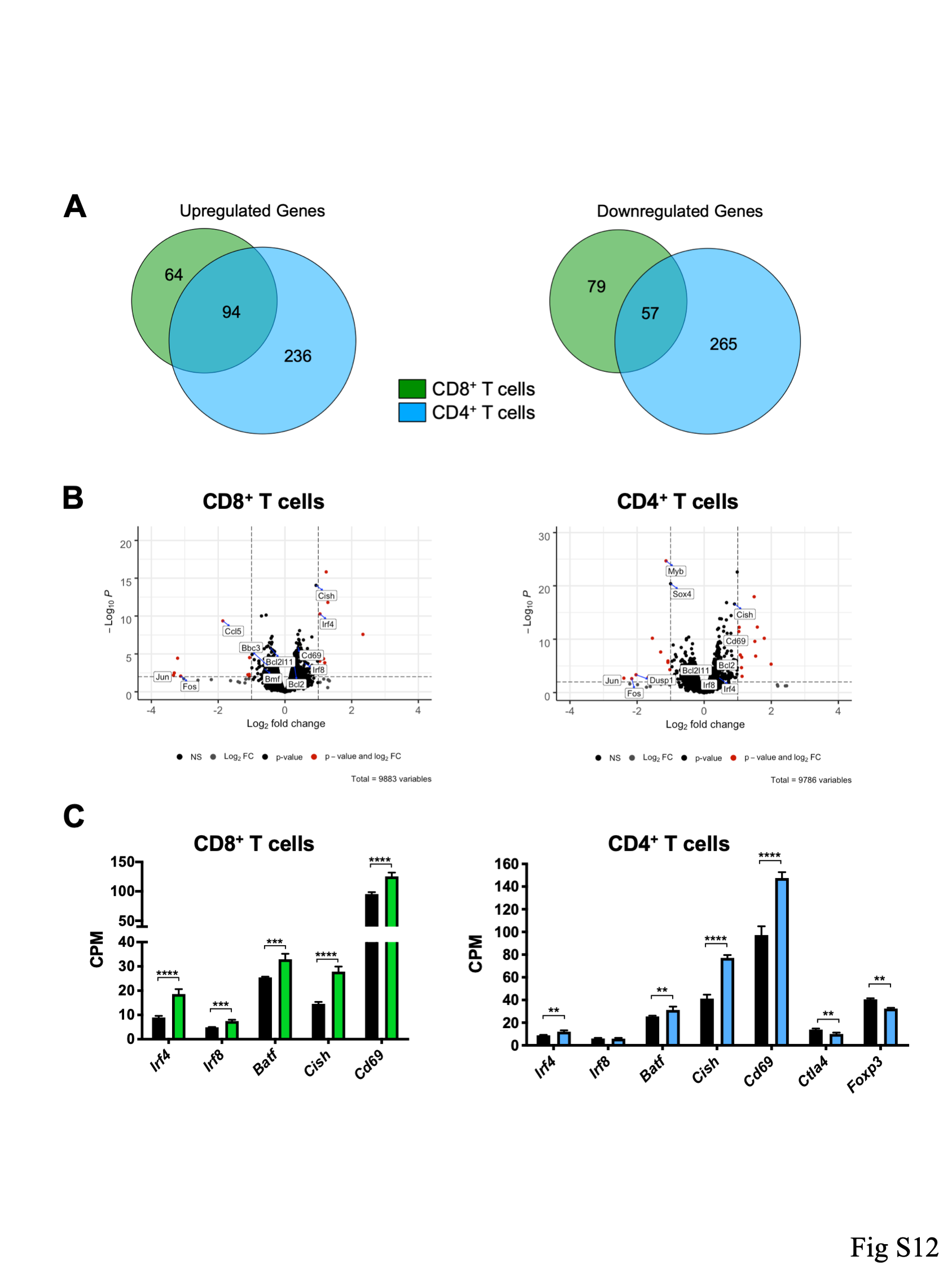

Supplement: Supplementary file 13 — Fig S12 [file 41419_2021_4285_MOESM13_ESM.tif]

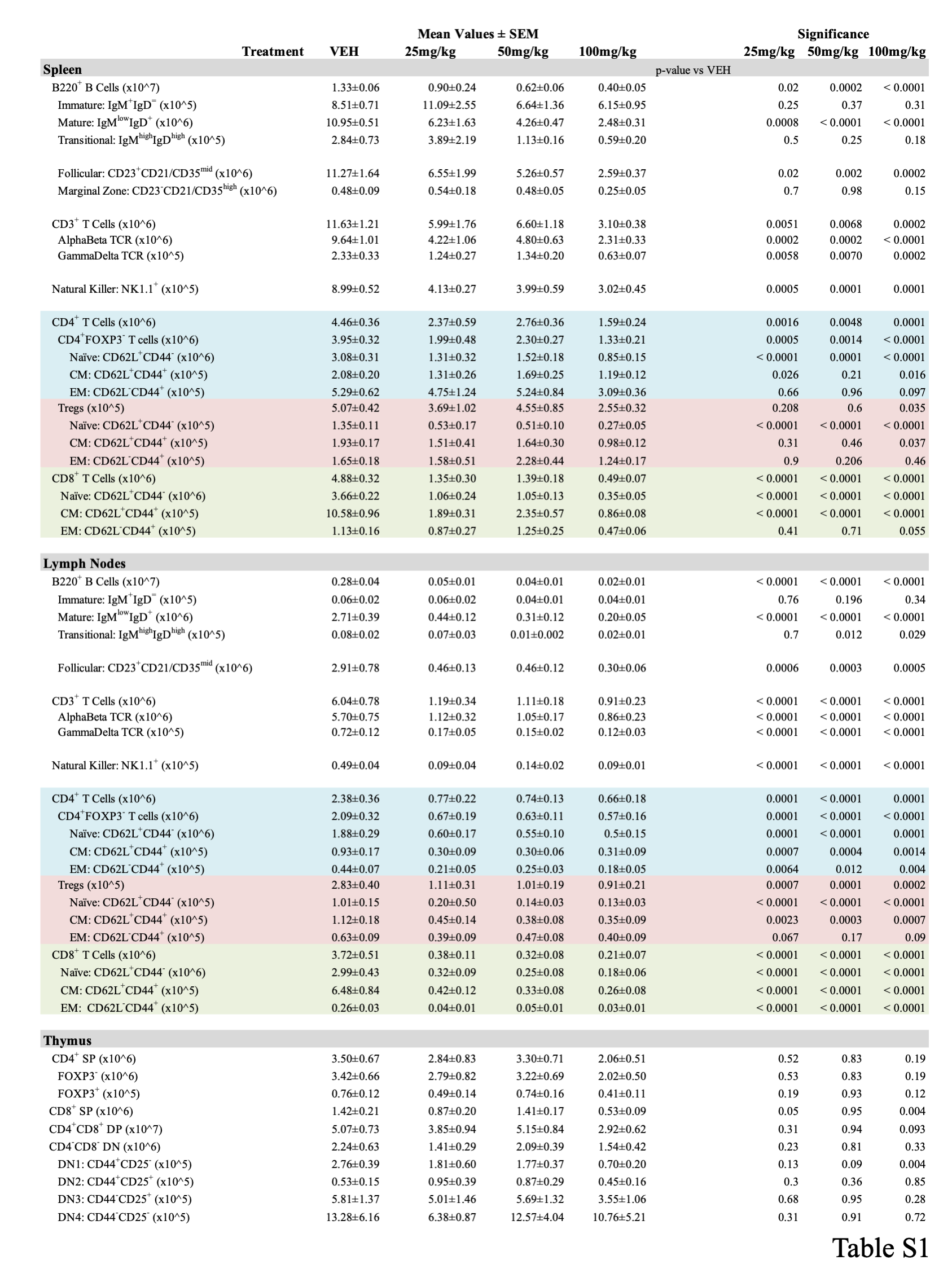

Supplement: Supplementary file 14 — Table S1 [file 41419_2021_4285_MOESM14_ESM.tif]

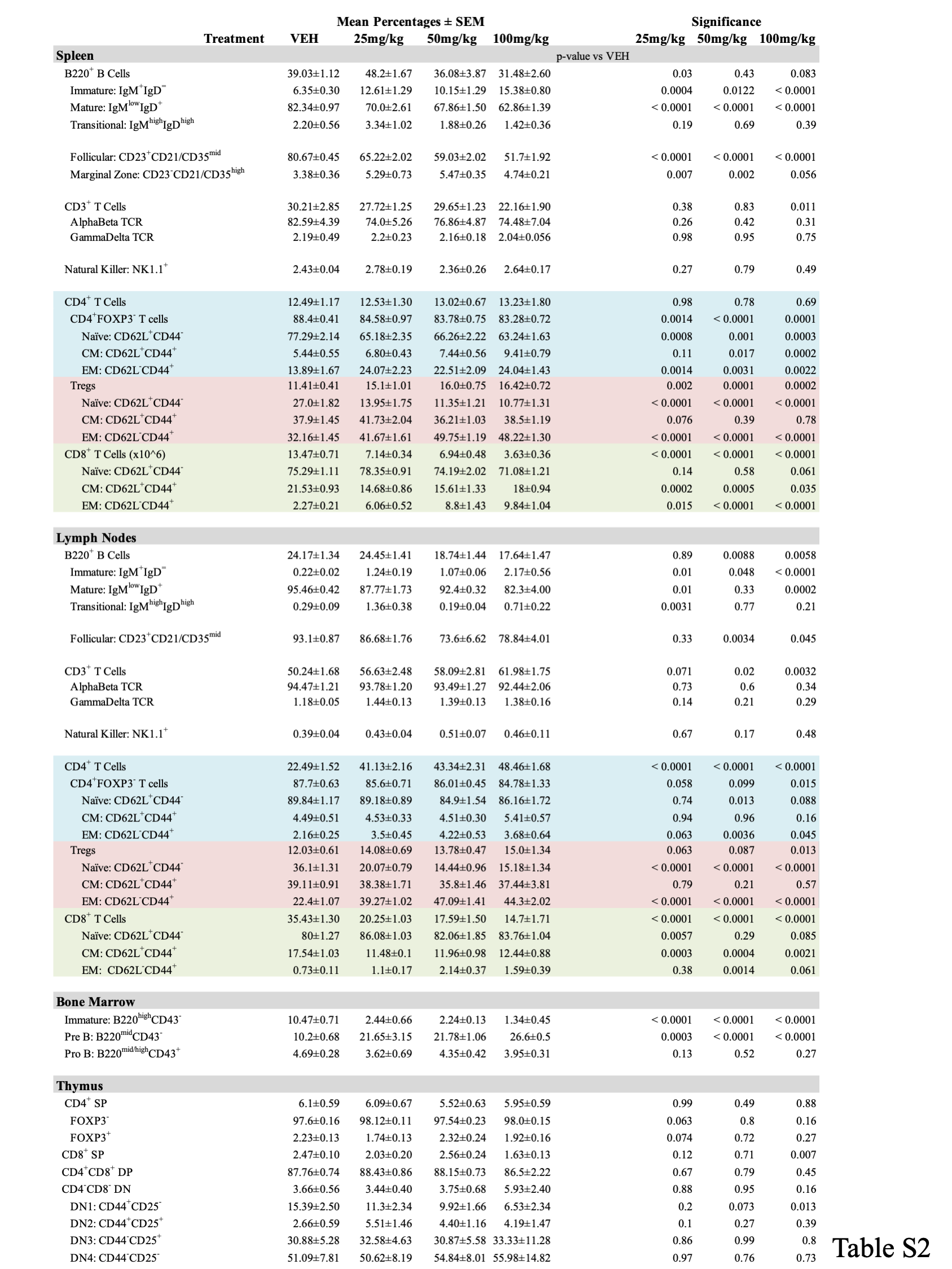

Supplement: Supplementary file 15 — Table S2 [file 41419_2021_4285_MOESM15_ESM.tif]
